# Cav3.2 T-type calcium channels shape electrical firing in mouse Lamina II neurons

**DOI:** 10.1038/s41598-019-39703-3

**Published:** 2019-02-28

**Authors:** Miriam Candelas, Ana Reynders, Margarita Arango-Lievano, Christoph Neumayer, Antoine Fruquière, Elsa Demes, Jawed Hamid, Céline Lemmers, Claire Bernat, Arnaud Monteil, Vincent Compan, Sophie Laffray, Perrine Inquimbert, Yves Le Feuvre, Gerald W. Zamponi, Aziz Moqrich, Emmanuel Bourinet, Pierre-François Méry

**Affiliations:** 1 Laboratories of Excellence - Ion Channel Science and Therapeutics, Montpellier, France; 2 Inserm U-1191, Montpellier, France; 3 CNRS UMR 5203, Institut de Génomique Fonctionnelle, Montpellier, France; 4 Université Montpellier, Montpellier, France; 5 Aix-Marseille-Université, CNRS, Institut de Biologie du Développement de, Marseille, France; 6 Department of Physiology and Pharmacology, University of Calgary, Calgary, Canada; 7 Plateforme de Vectorologie, Biocampus Montpellier, CNRS UMS 3426, INSERM US009, Montpellier, France; 8 CNRS, Institut des Neurosciences Cellulaires et Intégratives, Strasbourg, France; 9 UMR 5297, Interdisciplinary Institute for Neuroscience, Bordeaux, France

## Abstract

The T-type calcium channel, Cav3.2, is necessary for acute pain perception, as well as mechanical and cold allodynia in mice. Being found throughout sensory pathways, from excitatory primary afferent neurons up to pain matrix structures, it is a promising target for analgesics. In our study, Cav3.2 was detected in ~60% of the lamina II (LII) neurons of the spinal cord, a site for integration of sensory processing. It was co-expressed with Tlx3 and Pax2, markers of excitatory and inhibitory interneurons, as well as nNOS, calretinin, calbindin, PKCγ and not parvalbumin. Non-selective T-type channel blockers slowed the inhibitory but not the excitatory transmission in LII neurons. Furthermore, T-type channel blockers modified the intrinsic properties of LII neurons, abolishing low-threshold activated currents, rebound depolarizations, and blunting excitability. The recording of Cav3.2-positive LII neurons, after intraspinal injection of AAV-DJ-Cav3.2-mcherry, showed that their intrinsic properties resembled those of the global population. However, Cav3.2 ablation in the dorsal horn of Cav3.2^GFP-Flox^ KI mice after intraspinal injection of AAV-DJ-Cav3.2-Cre-IRES-mcherry, had drastic effects. Indeed, it (1) blunted the likelihood of transient firing patterns; (2) blunted the likelihood and the amplitude of rebound depolarizations, (3) eliminated action potential pairing, and (4) remodeled the kinetics of the action potentials. In contrast, the properties of Cav3.2-positive neurons were only marginally modified in Cav3.1 knockout mice. Overall, in addition to their previously established roles in the superficial spinal cord and in primary afferent neurons, Cav3.2 channel appear to be necessary for specific, significant and multiple controls of LII neuron excitability.

## Introduction

Pain therapeutics act at various levels of the nociceptive pathway and of the pain matrix where they frequently target ion channels, or receptors. The constitutive deletion of the Cav3.2 gene, encoding a T-type calcium channel, alleviates acute pain, inflammatory pain, and visceral pain in mice^1^, suggesting its utility as a therapeutic target. Accordingly, T-type calcium channel antagonists induce various forms of analgesia in animals and humans^2–4^. Thus, it is important to define 1) loci of action of Cav3.2 antagonists *in vivo*, and 2) the mechanism of action of Cav3.2 on cellular functions within these loci along the nociceptive tract.

Pharmacological data support pre- and post-synaptic roles for T-type channels in the dorsal horn (DH) of the spinal cord where primary sensory inputs are first integrated and processed^5–12^. Because of limitations concerning the pharmacology of T-type channels, genetic approaches were required in determining the role of Cav3.2 channels in primary afferent neurons^13–15^. A pre-synaptic facilitation of glutamatergic transmission in superficial spinal cord neurons, that is absent in Cav3.2 knockout mice, is strengthened in a model of painful diabetic neuropathy^10^. The intrathecal injection of Cav3.2 antisense shRNA blunts visceral pain^16^, has an anti-allodynic effect in a neuropathic pain model in rats^17^ and an anti-hyperalgesic effect in diabetic rats^18^. When restricted to Nav1.8-expressing primary afferent neurons, the conditional ablation of Cav3.2 also has a variety of effects, including a diminution in noxious cold perception, a reduction in chemical nociception and an anti-allodynic effect in chronic pain models^14^. Of note, the anti-allodynic effect of Cav3.2 deletion in non-myelinated fibers involves C-LTMR afferent fibers that project to lamina II, LII^14,19,20^. Thus, there is evidence for a role of Cav3.2 in the pathophysiology of primary sensory neurons. Post-synaptic Cav3.2 channels should be investigated within spinal cord networks as well, since Cav3.2 expression is found in lamina II-IV interneurons^13–15^. This region is well-known to receive descending nociceptive controls to gate sensory flow within the spinal cord, and to undergo disinhibition during allodynia^20–26^. Yet, some unidentified LII interneurons can express T-type channels^14,27^, and can exhibit T-type dependent activity^12,28–32^.

Here we show that Cav3.2 channels are broadly expressed in a heterogeneous population of LII neurons. An examination of the electrophysiological properties of unidentified neurons revealed a strong sensitivity of rebound depolarization to TTA-A2, a T-type channel inhibitor. Using a viral based cell identification strategy, we next focused on Cav3.2-expressing neurons, which exhibited electrophysiological properties close to those of the whole population. A Cre-recombinase induced deletion of Cav3.2 in adult mice changed the distribution of firing patterns, the properties of the action potential and eliminated the pairing of action potentials in LII neurons exhibiting a rebound behavior. Collectively, our findings reveal important functions for Cav3.2 in the processing of sensory information within the spinal cord.

## Materials and Methods

### Study approval

Animal procedures (#747) complied with the welfare guidelines of the European Community and were ethically approved by the Direction of Veterinary departments of Herault, France (Agreement Number A 34-172-41).

### Slice preparation for electrophysiological recordings

Adult 6–12 week-old wildtype or Cav3.2^GFP-Flox^ KI mice^14^ were anesthetized by injection of 0.1 ml/10 g ketamine (10 mg/ml) plus xylazine (1 mg/ml). They were perfused through the left ventricle with solution-1 [in mM; 122 N-Methyl-D-glucamine-Cl, 2.5 KCl, 1 CaCl_2_, 7 MgCl_2_, 2.5 NaHCO_3_, 1.5 NaH_2_PO_4_, 10 HEPES, 25 glucose, 0.2–5 ascorbic acid, 0.2–5 thiourea; pH 7.4, 0–2 °C, gassed with O_2_]^33^. N-Acetyl-L-cysteine 2 mM was added when studying mice aged 10–12 weeks. The lumbar region of the spinal cord was quickly removed in solution-1, embedded into low-melting agarose and 300µm-thick frontal sections were processed on a microtome (Leica VT12000S, Leica Microsystem, Nanterre, France) within 20 min and kept at least for 1 hour in solution-2 [in mM; 115 NaCl, 2.5 KCl, 2 CaCl_2_, 4 MgCl_2_, 26 NaHCO_3_, 1.25 NaH_2_PO_4_, 25 glucose, 0.2 ascorbic acid, 0.2 thiourea; pH 7.4, 30 °C, gassed with 95% CO_2_-5% O_2_].

### Patch-clamp recordings

Slices were immobilized with a nylon grid in a submersion chamber on the stage of an upright microscope (Olympus BX51WIF, Olympus Fr., Rungis, France) and superfused with solution-3 [in mM; 125 NaCl, 2.5 KCl, 2 CaCl_2_, 1 MgCl_2_, 26 NaHCO_3_, 1.25 NaH_2_PO_4_, 11 glucose; pH 7.4, 32 °C, gassed with 95% CO_2_-5% O_2_] at 2 ml/min for at least 15 min. Infrared differential interference contrast illumination was used to visualize neurons, with a x40 immersion objective and Nomarski differential interference contrast optics, and the images captured with a camera (Jenoptik ProgRes MF, Bayeux, France). When appropriate, the mCherry fluorescent signal was superimposed with the IR-DIC image in order to locate the Cav3.2-expressing neurons. Borosilicate glass pipettes were connected to the head stage of a Axon MultiClamp 700B, interfaced with an Axon Digidata 1550A and controlled with pClamp10 (all from Molecular Devices, Sunnyvale, USA). Drugs were either bath-applied or puff-applied locally. When bath-applied, solutions were changed by switching the supply of the superfusion system. When puff-applied in the vicinity of the neurons with a pipette, compounds were diluted in a HEPES-based medium containing in mM: 138 NaCl, 2.5 KCl, 2 CaCl_2_, 1 MgCl_2_, 3 NaHCO_3_, 1.25 NaH_2_PO_4_, 10 HEPES, 12 glucose, pH 7.4 with NaOH. Atto-dye 550 or Atto-dye 488 (Invitrogen, Waltham, USA) were occasionally included in the ejection pipette as a control. Slices were discarded after being exposed to a compound. All chemicals were obtained from Sigma-Aldrich (L’isle d’Abeau, France) excepted TTA-A2 kindly provided by Merck Research Labs (West Point, PA, USA).

For action potential recordings and for glutamatergic currents recordings, pipettes (6–8 MΩ) were filled with (in mM): 139 K-gluconate, 10 HEPES, 0.1 EGTA acid, 1 MgCl_2_, 2 MgATP, 0.5 Na-GTP, 5 Na_2_-phospocreatine, 2.5 Na-pyruvate, 2 malate, pH 7.3 with KOH (295 mOsm adjusted with KMeSO_3_). Atto-dye (550 or 488) was included in order to verify the identity of the neuron. Action potentials and resting properties were elicited by increasing and decreasing 2s-duration current pulses, respectively, from a holding adjusted at −70 mV^34^. Subthreshold electrical activity was triggered from −90 mV for 3 s to step-by-step current increments of 1.5 s until electrical activity was elicited^32^. Miniature glutamatergic currents were recorded in the presence of extracellular tetrodotoxin (TTX) 500 nM (Latoxan) at a holding potential of −70 mV. Analysis were performed with Axograph^35^ or Clampex10 (Molecular Devices) using either a threshold based detection or an appropriate template.

This procedure was adapted for the recordings of T-type currents and for GABAergic current recordings. The internal solution was (in mM)^36^: 125 CsMeSO_3_, 10 HEPES, 5 EGTA acid, 1 MgCl_2_, 2 MgATP, 0.5 Na-GTP, 5 Na_2_-phospocreatine, 2 Na-pyruvate, 2 malate, 7 KCl, pH 7.4 with CsOH (295 mOsm adjusted with CsMeSO_3_). When recording T-type currents, the external medium also contained TTX (500 nM), 4-aminopyridine (4-AP, 1 mM), tetra-ethyl-ammonium (TEA, 10 mM) and ivabradine (1 µM). For current-voltage relationships, neurons were voltage-clamped at −90 mV and 1s-duration pulses of increasing amplitude were delivered by steps of 5 mV. For the voltage ramp protocol, after a 500 ms step at −100 mV, the voltage was increased linearly to 40 mV during 200 ms. When recording miniature GABAergic currents, the external solution was supplemented further with APV (50 µM), DNQX (30 µM) and neurons were voltage-clamped at 20 mV.

### Plasmid cloning and virus production

The minimal Cav3.2 promoter used was based upon nucleotide sequence derived from mouse CACNA1H gene locus sequence (ENSMUSG00000024112). We purchased a mouse Cav3.2 promoter clone (MPRM 12993) from promoter clone library (GeneCopoeia) with additional restriction endonuclease sites Mlu I and BamHI at the 5 prime and 3 prime ends, respectively. This clone consists of 1462 nucleotides of the mouse Cav3.2 gene, with 1450 nucleotides 5′ to Exon 1 and 12 nucleotides of the non-coding exon 1 of mouse Cav3.2 gene (ENSMUSG00000024112, see Supplementary Fig. S1). This sequence was subjected to online analysis for core promoter sequences (http://gpminer.mbc.nctu.edu.tw/index.php) to confirm the presence of TATA and CCAAT boxes as identified in the rat sequence^37^. This promoter fragment was isolated via restriction with MluI and BamHI from the original promoter clone (MPRM 12993) and sub cloned into pAAV-hSyn-hChR2(H134R)-mcherry (Addgene26976) using the same restriction sites (MluI and BamHI) to replace the hSyn promoter sequences with mouse Cav3.2 promoter sequence. The plasmid pAAV-Cav3.2-mCherry was cloned using pAAV-Cav3.2-hChR2-mCherr*y*. A 1300 bp fragment was amplified by PCR with the forward primer ATTCTAGAAAGACGAAGCCGAGGC and the reverse primer AAGAATACCAGTCAATCTTTCACAA. The PCR product was cloned into pAAV-Cav3.2-hChR2-mCherry using the XbaI and AfeI restriction sites. The pAAV-Cav3.2-Cre-mCherry was obtained by cloning the mCherry-IRES-Cre sequence of the plasmid pAAV-EF1a-mCherry-IRES-Cre (Addgene 55632) into the vector pAAV-Cav3.2-mCherry using BamHI and EcoRI restriction sites.

The Viral production in HEK-293 cells was performed with the DJ-packaging system (Cell Biolabs) and purified on a heparin column according to (Morgenstern, Marongiu *et al*.^38^). Virus titers (>10^11^ copies/ml in the present study) were estimated by q-PCR using either the forward primer TTGTTCTCCACCTCCTTC and the reverse primer GCAACATAGCAACCTCAG ACC, or the forward primer ACTGTGTTTGCTGACGCAAC and the reverse primer AGCGAAAGTCCCGGAAAG.

### Intraspinal injection of virus

Mice were anesthetized with ketamine plus xylazine and placed on a heating sheet (37 °C). A 2cm-long incision was performed at the level of the L1 vertebra. The spinal cord was exposed by removing the dorsal portion of the vertebra L1 using a laminectomy forceps^39^ or by carefully bending the spine without further surgery^40^. A micro syringe held on a stereotaxic frame and filled with the virus suspension was positioned at the rostral-most portion of the exposed spinal cord, 500 µm laterally with respect to the posterior median sulcus. Injections (800 nl) were performed at a depth of 150–300 µm, at a rate of 100–200 nl/min. After careful retraction of the syringe, this operation was repeated at the caudal-most portion of the exposed spinal cord. The fascia and the skin were sutured before the mouse was transferred into a recovery cage for >4 hours, and postsurgical treatments were provided according to guidelines. This procedure did not increase inflammatory markers, 3–4 weeks after injection, as assessed by changes in the morphologies of astrocytes and microglial cells^39,40^.

### Immunofluorescence experiments

Mice (8–14 weeks) were anesthetized with ketamine plus xylazine and processed as follows^14^. Spinal cord were fixed with a transcardiac perfusion of cold PBS containing PFA 4% and a post-fixation (<2 hours) at 4 °C. They were embedded in 4% agarose and sectioned at 30–40 µm with a vibratome (Microm HM 650 V, Brignais, France). The sections were blocked with TBS plus 0.05% tween 20 (TBST), 0.2% Triton X 100 and 10% normal serum (goat or donkey), 22 °C, for 1 hr; the incubations with primary antibodies (Supplementary Table 1) were done in blocking solution at 4 °C overnight; and the incubation with secondary antibodies (Supplementary Table 1) were performed at 22 °C, 2 hrs. They were included in Dako Mounting Medium (Agilent Tech., Santa Clara, USA) and images were performed on a confocal microscope (Leica SP8-UV, Nanterre, France) with x40 (Plan Apochromat 1.3 NA oil DIC) and x63 (Plan Apochromat 1.4 NA oil DIC) objectives. Image processing was performed with FiJi^41^, and cell densities were normalized to DAPI labelling or to the appropriate antigen as stated in the text.

### *In situ* hybridization and immunostaining

*In situ* hybridization (ISH) and immunostaining were performed according to Moqrich *et al*.^42^. For ISH, all the probes were designed as follows: (i) Transcript-specific regions were determined using Blastn (NCBI) software and specific primers (Supplementary Table 2) were designed accordingly using PrimerBlast (NCBI) software. (ii) One first PCR was performed using the GoTaq Hot-Start polymerase kit (Promega) and cDNA templates from adult mouse dorsal root ganglia (for Cav 3.2 and 3.3) or hippocampus (for Cav3.1). (iii) A second PCR was performed, in which the product of the first PCR was used as template and the T7 RNA polymerase promoter sequence was fused to the reverse primer. iv) RNA antisense probes were synthetized using the T7 RNA polymerase (Promega) and dioxygenin (DIG)-labelling system from Roche. Mice were deeply anesthetized with a mix of ketamine/xylazine and then transcardially perfused with an ice-cold solution of 4% PFA in PBS. After dissection, spinal cords were post-fixed for 12 h at 4 °C and transferred into a 30% (w/v) sucrose solution for cryoprotection before being embedded in Optimal Cutting Temperature compound (Leica), then snap-frozen on dry ice and stored at −80 °C. Sixteen to 18 µm cryosections were obtained using a standard cryostat (Leica). Probes were hybridized overnight at 55 °C, and the slides incubated with the horseradish peroxidase anti-digoxigenin antibody (Roche). Final detection was achieved using Cy3 TSA plus kit (Perkin Elmer). Sections were then incubated overnight at 4 °C with the appropriate antibody (anti-RFP for mCherry, or rabbit anti-PKC_ϒ_), diluted in PBS supplemented with 10% (v/v) donkey serum (Sigma), 3% (w/v) bovine albumin (Sigma) and 0.4% (v/v) Triton X-100. Anti-rabbit Alexa Fluor 488 or anti-rabbit Alexa Fluor 647 antibodies (Supplementary Table 1) were used for secondary detection. Nuclei were stained with Hoechst (Sigma Aldrich). Slides were mounted with Immu-Mount (Thermo Scientific) prior to observation under LSM780 confocal microscope (Carl Zeiss, Iena, Germany). Contrast was adjusted using Photoshop software.

### Statistics

Data were generally expressed as mean ± SEM. In few cases, families of individual values are shown. They were compared with the appropriate tests using Prism, and p < 0.05 was considered as significantly different. The numbers of neurons and mice studied using the patch-clamp techniques are summarized in Supplementary Tables 3 and 4.

## Results

### Distribution of cells expressing Cav3.2-GFP and neurochemical phenotype

**Figure 1:**
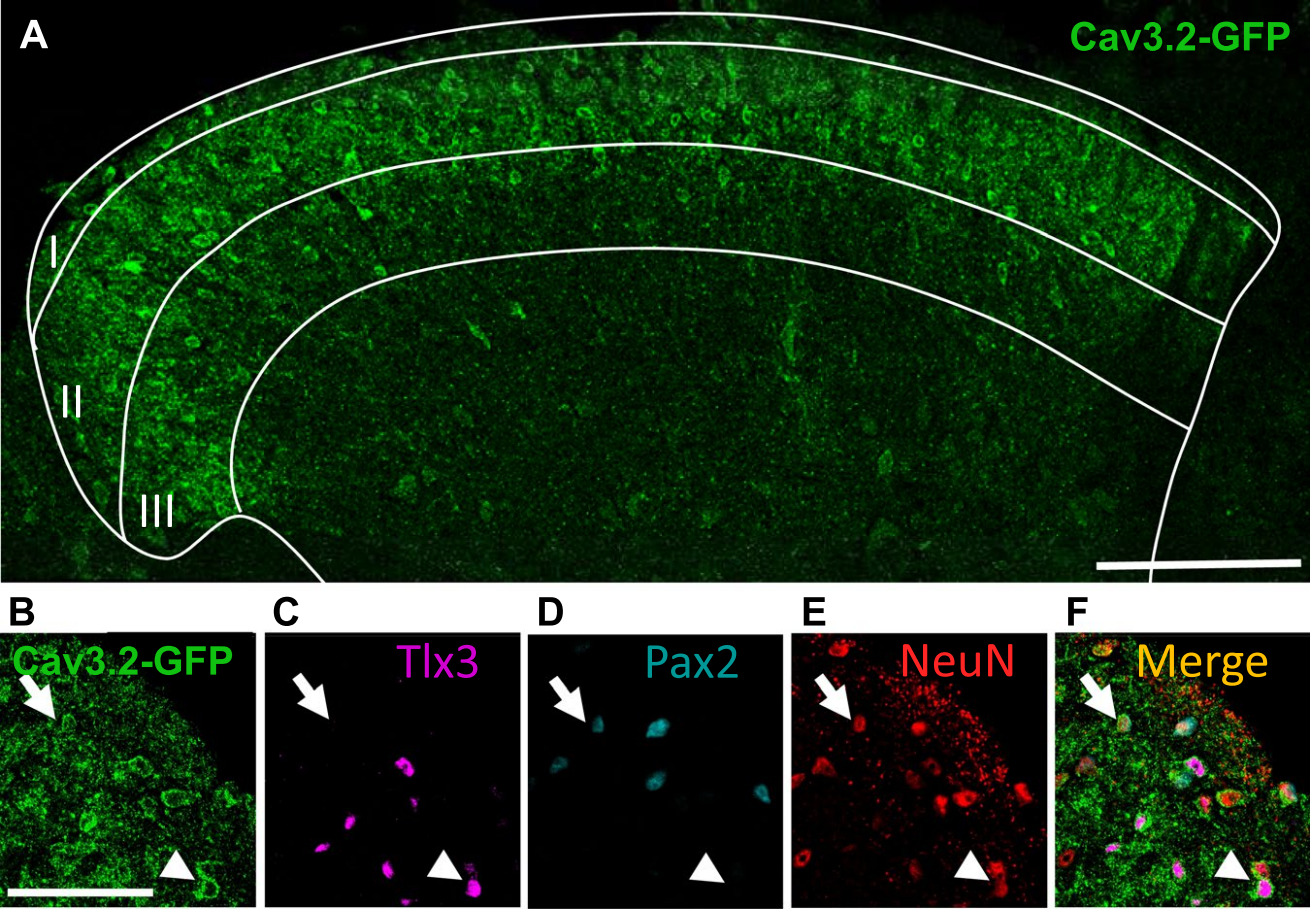
GFP immunofluorescence in the spinal cord of the adult Cav3.2^GFP-Flox^ KI mouse. (**A**) Immunostaining for GFP (green), showing the localization of Cav3.2-GFP expressing cell bodies and neuronal fibers in the dorsal horn of the lumbar spinal cord. The majority of GFP is found in the upper layers of the dorsal horn. Coexpression of GFP ((**B**) green) with either Tlx3 ((**C**) purple, arrow), or Pax2 ((**D**) blue, arrowhead) and NeuN ((**E**) red), as well as the superimposition of the four images (**F**). Scale bars: (**A**) (100 µm), (**B**–**F**) (25 µm).

To determine the nature of Cav3.2-expressing cell types in the spinal cord, we took advantage of Cav3.2^GFP-Flox^ KI mice that express GFP at the Cav3.2 locus^13^. In adult Cav3.2^GFP-Flox^ KI mice, GFP labelled cell bodies and nerve terminals mostly located in LII of the spinal cord (Fig. 1A), as previously reported^14^. The majority of GFP-positive cell bodies (about 70%) co-expressed Tlx3, a marker of excitatory neurons (Fig. 1B,C). A small fraction (about 13%) of GFP-positive cell bodies were positive for Pax2, a marker of inhibitory interneurons (Fig. 1B,D)^43,44^. The neuronal origin of GFP-expressing cells in the spinal cord was further confirmed using pan-neuronal NeuN staining (Fig. 1B,E and Table 1). Overall, Cav3.2 was present in about 60% of the LII neurons, contributing to about half of the Tlx3-positive subset (47.5%, Table 1) and to a much smaller subpopulation of Pax2-positive neurons (12.6%, Table 1).

**Table 1:** Coexpression of GFP with neuronal markers in Cav3.2^GFP-Flox^ KI mice.

% of GFP-expressing neurons			% of neurons	
NeuN (8)	Tlx3 (15)	Pax2 (4)	Tlx3 (14)	Pax2 (3)
85.3 ± 5.2	70.8 ± 2.6	13.1 ± 4.7	47.5 ± 2.69	12.6 ± 6.1

Numbers are the mean ± SEM of GFP-expressing neurons. Number of mice are indicated within brackets.

**Figure 2:**
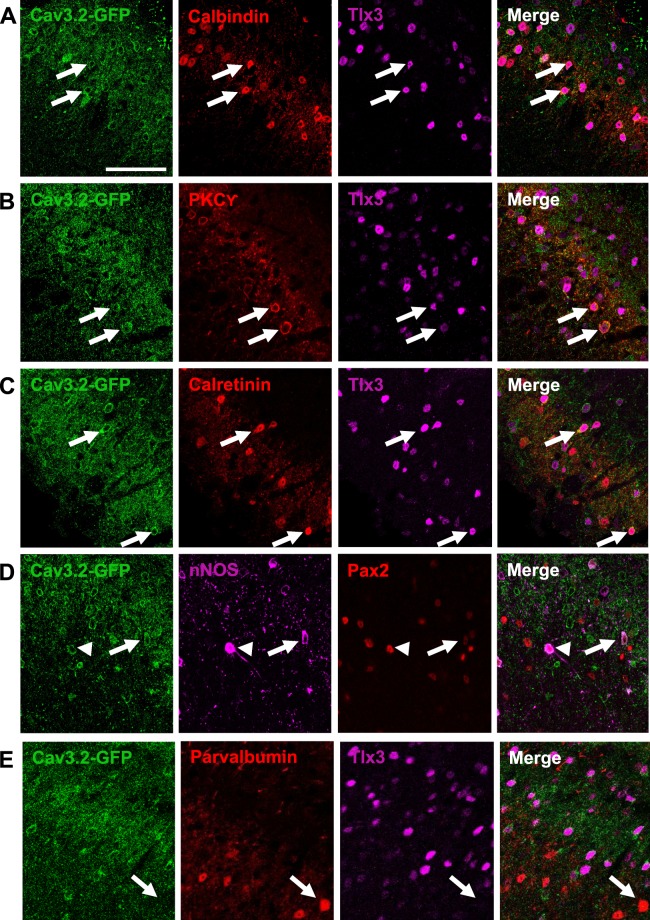
GFP labeled a heterogeneous population in the LII of Cav3.2^GFP-Flox^ KI mice. GFP (green) was coexpressed with calbindin (**A**), PKC_ϒ_ (**B**), calretinin (**C**), nNOS (**D**), but not with parvalbumin (**E**). These neurons were either excitatory, Tlx3-positive (arrows, (**A**–**C**,**E**)) and Pax2-negative (arrow, (**D**)); or inhibitory, Pax2-positive (arrowhead, (**C**)). Scale bar: 50 µm.

Further dissection of neuronal cell types expressing Cav3.2 using immunolabelling for GFP and known markers of subpopulations of LII-LIII neurons involved in sensory processing by the DH (calbindin, CB; protein kinase C gamma, PKC_ϒ_; calretinin, CR; neuronal NOS, nNOS; parvalbumin, PV), revealed a heterogeneous expression pattern (Fig. 2, Table 2). Most of GFP-expressing neurons were CB-positive (43.2% of GFP neurons, Table 2, Fig. 2A) or PKC_ϒ_-positive (37.0% of GFP neurons, Table 2, Fig. 2B), two markers of excitatory LII neurons^19,20,45,46^. The GFP-positive neurons expressing CB also expressed Tlx3 (40.3% of GFP neurons, Table 2), a marker of excitatory DH interneurons^43^. The GFP-positive neurons expressing PKC_ϒ_ were mainly excitatory since 85.7% of the PKC_ϒ_-positive neurons expressed GFP, PKC_ϒ_ and Tlx3 (Table 2). Interestingly, most PKC_ϒ_-expressing neurons, which are disinhibited during mechanical allodynia^23,24,26^, expressed Cav3.2 (91.7%, Fig. 2B, Table 2). Besides expressing Cav3.2, some PKC_ϒ_ interneurons could also express Cav3.1 and Cav3.3 mRNAs, as detected by *in situ* hydridization (Supplementary Fig. S2). Almost half of the CR neurons, which relay nociceptive inputs to projection neurons^23,24^, were GFP-positive (Fig. 2C, Table 2). This population was mostly excitatory, since CR labeling alone, and CR/Tlx3 double labelling represented 26.1% and 22.1%, of the GFP-positive neurons, respectively (Table 2). A small subset of GFP-expressing neurons also expressed nNOS (10.7%), and they accounted for almost half of the nNOS-positive neurons (42.1%, Fig. 2D, Table 2). These neurons, are both inhibitory and excitatory (Table 2), in agreement with a previous report^47^ and are differentially recruited during noxious stimulations^48^. In contrast, the PV-expressing neurons of LIII, some of which are gatekeepers of the gate control theory for mechanical allodynia^26^ were marginally GFP-positive (7.4% of the PV neurons, Fig. 2E, Table 2). Altogether, these results indicate that Cav3.2 channels are expressed in a wide variety of spinal cord neurons, which relay primary afferent activity to projection neurons during mechanical allodynia.

**Table 2:** Coexpression of GFP with markers of the LII in adult Cav3.2^GFP-Flox^ KI mice mice.

CB	CB/Tlx3	PKC_ϒ_	PKC_ϒ_/ Tlx3	CR	CR/Tlx3	nNOS	nNOS/ Pax2	PV
**% of GFP-positive neurons**								
45 ± 5.5 (n = 5)	40.2 ±5.1 (n=5)	39.7 ± 5.6 (n = 6)	43.0 ± 4.2 (n = 4)	22.5 ± 5.7 (n = 5)	22 ± 4 (n=4)	10.7 ± 2.8 (n = 4)	1.8 ± 1.8 (n = 2)	1.6 ± 1.1 (n = 3)
**% of neurons**								
46.25 ± 5 (n = 5)	69.37 ± 7.4 (n = 5)	89.5 ± 2.2 (n = 6)	85.7 ± 1.3 (n = 4)	41.1 ± 8.5 (n = 5)	36.1 ± 6.4 (n = 4)	42.1 ± 14.4 (n = 4)	20.3 ± 20 (n = 2)	7.6 ± 1.1 (n = 3)

Numbers are the mean ± SEM of GFP-expressing neurons. Number of mice are indicated within brackets. Counts for nNOS, calretinin (CR) and calbindin (CB) were limited to LII, while they also included the outer part of LIII in the case of PKC_ϒ_ and parvalbumin (PV).

### Effects of T-type channel inhibition on synaptic transmission

**Figure 3:**
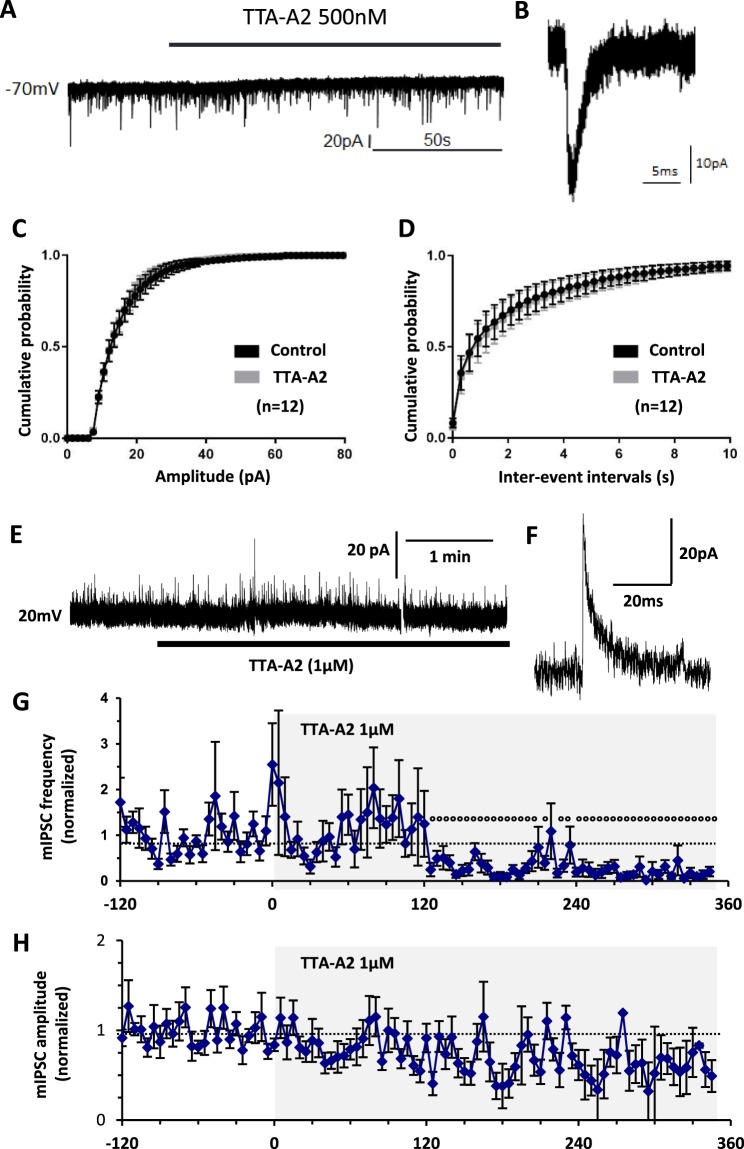
Effects of T-type calcium channel inhibitors on neurotransmission of LII neurons in the spinal cord of adult mice. (**A**) Typical whole cell patch clamp recording, performed in LII of an acute 300µm-thick slice taken from a lumbar segment of an adult mouse, showing miniature excitatory postsynaptic currents (mEPSC) of a neuron held at −70 mV. (**B**) Trace showing an individual mEPSC. (**C**,**D**) Mean cumulative distribution of the amplitudes (**C**) and the inter-event intervals (**D**) of mEPSC in the absence (control) or presence of 500 nM TTA-A2 (n = 12). (**E**) Typical whole cell patch clamp recording, performed in LII of an acute 300µm-thick slice taken from a lumbar segment of an adult mouse, showing miniature inhibitory postsynaptic currents (mIPSC) of a neuron held at 20 mV. (**F**) Trace showing an individual mIPSC. (**G**,**H**) Mean frequencies (**G**) and amplitudes (**H**) of mIPSC in the absence and presence of 1 µM TTA-A2 ejected at time 0, as shown by the grey area (n = 12). p < 0.05, paired student’s t-test, is indicated as dots above symbols. Data were normalized to the mean of all the values recorded before time 0 (solid lines). At time 0, the raw frequency was 0.59 ± 0.17 Hz, and the raw amplitude was 17.4 ± 1.6 pA.

The Cav3.2-expressing primary afferent neurons project to internal LII where most Cav3.2 neurons are found^14^. To study the T-type channel contribution to neurotransmission, we examined the synaptic currents in acute spinal cord slices from adult mice using whole cell patch clamp. Miniature excitatory post-synaptic currents (mEPSCs) of LII neurons were recorded at a membrane potential of −70 mV^49^, in the presence of a sodium channel blocker (TTX 500 nM, Fig. 3). Addition of the T-type blocker TTA-A2 (500 nM) in the superfusion medium did not modify the mEPSCs in the experiment of Fig. 3A,B. On average, TTA-A2 had no effect of the mean distributions of either the amplitudes or the inter-event intervals of mEPSCs (n = 12, Fig. 3C,D). Another T-type channel blocker, NiCl_2_ (100 µM) also did not change the mean glutamatergic neurotransmission (Supplementary Fig. S3A,B). In addition, TTA-A2 did not significantly modify the spontaneous mEPSCs over time in the continuing presence of the HCN inhibitor ivabradine (1 µM, n = 9, Supplementary Fig. S3C,D) or under constant GABA_A_-ergic (Gabazine 3 µM) and Glycinergic (strychnine 3 µM) receptor inhibition (n = 5, Supplementary Fig. S3E,F). In contrast, T-type channel blockade homogeneously inhibited the spontaneous GABAergic currents of LII neurons (Fig. 3E–H), as shown in a typical experiment were the puff application of TTA-A2 (1 µM) altered the frequency of the miniature GABAergic currents recorded at 20 mV (Fig. 3E). On average, inter-event intervals (Fig. 3G), but not amplitudes (Fig. 3H), of GABAergic miniature currents were significantly diminished in the continuing presence of TTA-A2. Therefore, T-type channels participated in spontaneous inhibitory synaptic transmission, but not in the excitatory transmission of LII neurons. This does not exclude the possibility that T-type channels might exert significant contributions in some subpopulations of LII neurons^11^.

### Association of T-type channel activity with firing patterns of LII neurons

T-type calcium channels participate in the intrinsic properties of some types of DH neurons^12,29,30,32^. Accordingly, voltage ramps or current-voltage relationships demonstrated the presence of low-voltage and high-voltage activated calcium currents in LII neurons, which could be, respectively, inhibited by NiCl_2_ and CdCl_2_ (Supplementary Fig. S4). Therefore, we analyzed the firing properties of LII neurons elicited by current pulses from a holding potential of −70 mV in acute lumbar slices from adult mice (Supplementary Figs S5 and S6). The observed firing patterns were highly heterogeneous, with delayed firing profiles being the most frequent (28.5%), and various proportions of transient (18%), irregular tonic (16.5%), regular tonic (12.8%), gap firing (12.7%) and single spiking (11%) (n = 134, Supplementary Fig. S5C).

**Figure 4:**
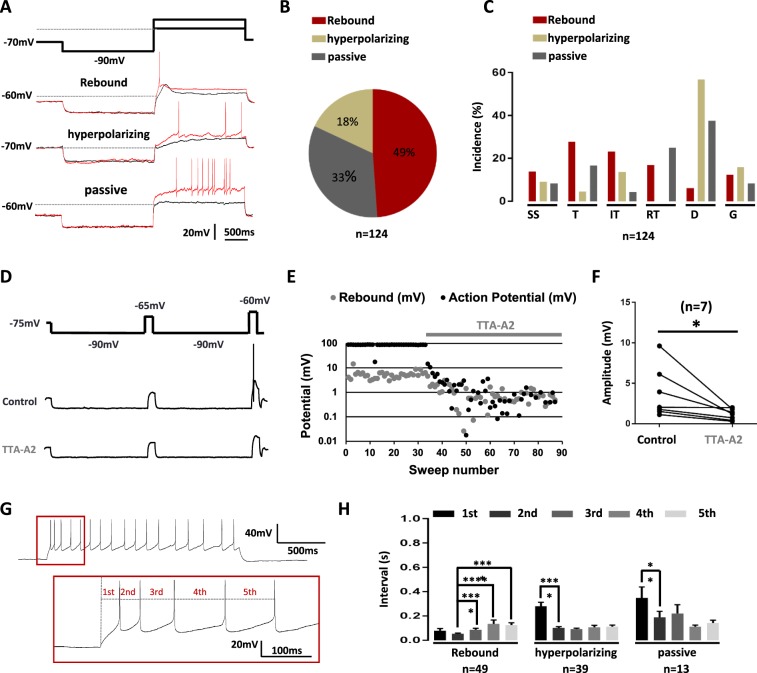
The rebound induced by hyperpolarization is T-type channel dependent. (**A**) A 2-step current clamp protocol with a hyperpolarizing prepulse to −90 mV induced three kind of responses upon step by step depolarizations: rebound shaped, hyperpolarizing kinetics and passive depolarizations. Red traces show the firing at the rheobase. (**B**) Proportion of LII neurons exhibiting these three subthreshold activities. (**C**) Relationships between firing patterns and subthreshold behaviors. SS (Single spiking), T (Transient), IT (Irregular Tonic), RT (Regular Tonic), D (Delayed) and G (Gap). (**D**) Multi-steps protocol used in eliciting subthreshold (first depolarizing step) and supra-threshold activities (second depolarizing step), and typical recordings in the absence (control) and presence of TTA-A2. (**E**) Quantification of the effects of TTA-A2 puff application on the amplitude of the subthreshold and supra-threshold activities in a typical experiment in a LII neuron. (**F**) In all similar experiments, TTA-A2 (1 µM) diminished the subthreshold rebound depolarization. (**G**) The intervals of the 5 first action potentials were quantified in LII neurons. (**H**) Mean intervals showed a pairing of the first two action potentials in neurons exhibiting rebound subthreshold activity. Bars are the means and lines are the SEM ****p < 0.0001; ***p < 0.001; **p < 0.01; *p < 0.05 using a Wilcoxon paired test.

Subthreshold depolarizations elicited from a hyperpolarizing conditioning prepulse at −90 mV were more homogeneous since three behaviors were observed: rebound (49%), hyperpolarizing (18%) and apparently passive decays (33%) (n = 124, Fig. 4A,B). Although these properties did not tightly segregate with a firing pattern (n = 124, Fig. 4C), neurons exhibiting rebounds more likely supported higher activities at the onset of the pulse, namely single spiking, transient and irregular tonic patterns. Since rebound shaped depolarizations can be a signature of T-type channel activity^50^, a multiple step protocol including a subthreshold and a suprathreshold current injection was designed to investigate the role of T-type channels on neuronal firing (Fig. 4D). When a neuron elicited a rebound depolarization, puff application of the T-type channel inhibitor TTA-A2 (1 µM) significantly decreased the rebound amplitude during the first subthreshold step and abolished action potential firing during the second suprathreshold step (n = 7, Wilcoxon paired test, p = 0.01, Fig. 4E,F). Rebound depolarization was not only associated with an “all-or-none” effect on firing. Indeed, an analysis of the first five action potentials revealed that neurons exhibiting rebound depolarizations also specifically exhibited a pairing of the first two action potentials, (Fig. 4G,H), the inter-event interval of the second action potential being shorter than all others (n = 49, Wilcoxon paired tests, Fig. 4H). In neurons with a hyperpolarizing decay (n = 39) and neurons with a passive response (n = 13), the first action potential had the longest latency (Wilcoxon paired tests, Fig. 4H), and the next action potentials occurred regularly. Altogether, these data not only confirm the involvement of T-type calcium channels in the spinal cord, but show that they are mandatory for specific firing patterns.

### Properties of Cav3.2-expressing neurons in LII of the spinal cord

**Figure 5:**
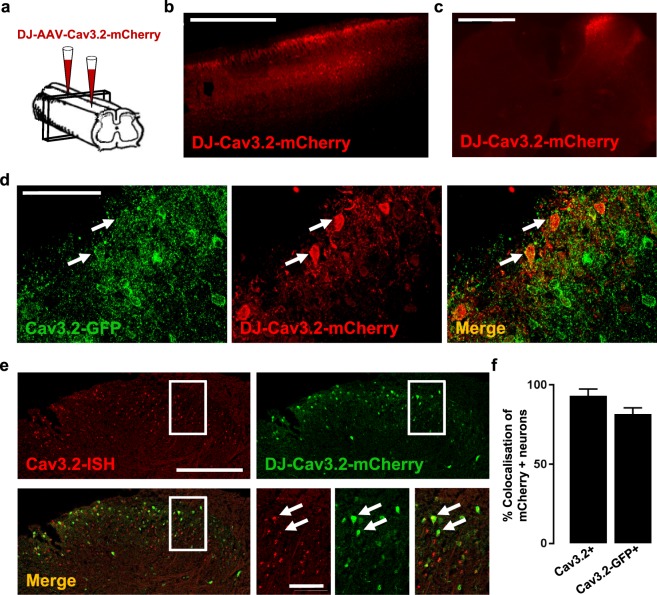
Identification of Cav3.2-expressing neurons in the lumbar spinal cord in adult mice. (**A**) Schema of the injection protocol, adapted from Inquimbert *et al*.^39^. (**B**,**C**) Detection of mCherry expression in a parasagittal (**B**) and a frontal (**C**) spinal cord slice three weeks after intraspinal injections of AAV-DJ-Cav3.2-mCherry virus in wildtype mice. (**D**) Detection of GFP (green) and mCherry (red) expressions by immunofluorescence in a frontal spinal cord section three weeks after intraspinal injections of AAV-DJ-Cav3.2-mCherry virus in a Cav3.2^GFP-Flox^ KI mouse. Co-expression is indicated (arrows). (**E**) Detection of Cav3.2 mRNA expression (red) by *in situ* hybridization and of mCherry expression (green) by immunofluorescence in a frontal spinal cord section three weeks after intraspinal injection of AAV-DJ-Cav3.2-mCherry virus in a wildtype mouse. Co-expression is indicated (arrows). (**F**) Mean colocalizations between mCherry-positive neurons and either Cav3.2 mRNA in wildtype mice or GFP in Cav3.2^GFP-Flox^ KI mice. Bars are the means and lines are the SEM from 2 and 4 mice in immunofluorescence and *in situ* hybridization experiments, respectively. Scale bars: **B** (1 mm), **C** (500 µm), **D** (50 µm), **E** (200 µm, 50 µm).

Since the excitability of LII neurons appeared to be dependent on T-type calcium channels, a selective role for Cav3.2 was then investigated in greater detail. A 1.4 kb fragment of the Cav3.2 promoter gene was inserted into a pAAV expression vector in order to control the selective expression of the fluorescent protein mCherry in Cav3.2-expressing neurons. The viral particles were injected at two sites of the lumbar spinal cord in 4 weeks-old wildtype mice (Fig. 5A). Detection of mCherry-positive neurons by immunofluorescence 3–4 weeks after injection showed a large coverage of the lumbar segments in the rostrocaudal direction (Fig. 5B) that was specific for the ipsilateral side (Fig. 5C). The mCherry-positive neurons were also GFP-positive, after injection of the AAV-DJ-Cav3.2-mCherry virus in a Cav3.2^GFP-Flox^ KI mouse (Fig. 5D). Similarly, the mCherry-positive neurons detected by immunofluorescence expressed Cav3.2 mRNA, as detected by *in situ* hybridization, after injection of the AAV-DJ-Cav3.2-mCherry virus in a wildtype mouse (Fig. 5E). On average, the Cav3.2-promoter driven expression of mCherry was found in 82% of the Cav3.2-GFP positive neurons, and in 93% of the Cav3.2 mRNA-expressing neurons (Fig. 5F). Thus, it faithfully reported Cav3.2 expression with a bright fluorescence signal.

**Figure 6:**
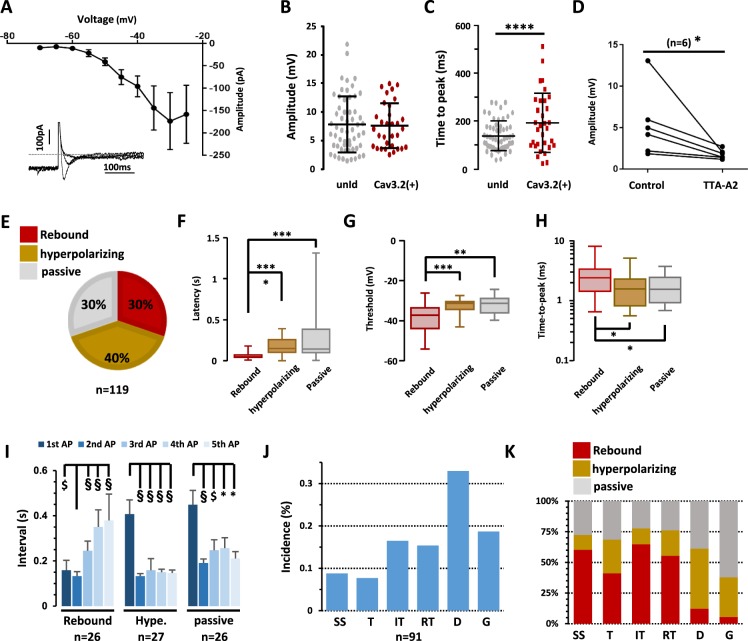
Electrophysiological properties of Cav3.2-expressing neurons in the LII of the spinal cord of adult mice. (**A**) Mean current-voltage relationship of a low-voltage activated calcium current in Cav3.2-expressing neurons. Symbols are the means and lines are the SEM from 7 LII neurons. ***Inset***, typical inward currents evoked from −90 mV to −60, −50 and −40 mV. The extracellular medium contained 100 µM CdCl_2_. (**B**) Amplitude and (**C**) time to peak of the rebound shaped depolarisations in Cav3.2-mCherry neurons (n = 32) as compared to unidentified neurons (n = 57). Lines are means and SEM, and dots are the individual values. ****p < 0.001 using a Mann-Whitney test. (**D**) 1 µM TTA-A2 inhibited the rebound-shaped subthreshold depolarisation of LII Cav3.2-expressing neurons. *p = 0.01 using a Wilcoxon paired test. (**E**) Proportions of the subthreshold properties of Cav3.2-expressing neurons. (**F**–**H**) Box and whiskers representations of action potential latency (**F**), threshold (**G**), and time-to-peak (**H**) in Cav3.2-mCherry neurons exhibiting rebounds (n = 32), hyperpolarizing- (n = 28) and passive-depolarisation profiles (n = 33), as indicated. ***p < 0.005; **p < 0.01; *p < 0.05, using a Mann Whitney test. Action potential properties were analysed as in Fig. S3, and kinetics of depolarisations were triggered as in Fig. 4A. (**I**) Mean intervals showed a selective pairing of the first two action potentials in Cav3.2-expressing neurons with rebound subthreshold activity. Bars are the means and lines are the SEM Significances are indicated as: ^§^p < 0.001; ^$^p < 0.005; *p < 0.05, using a Wilcoxon paired test. (**J**) Firing patterns of LII mCherry-positive neurons from AAV-DJ-Cav3.2-mCherry injected mice recorded in the current clamp mode of the whole-cell patch clamp technique (as in Fig. S3). (**K**) Relationships between firing patterns and subthreshold behaviors. SS (Single spiking), T (Transient), IT (Irregular Tonic), RT (Regular Tonic), D (Delayed) and G (Gap), see Fig. S3 for explanations. Dots are the means and lines are the SEM.

Having established and validated a novel tool for identifying Cav3.2-expressing neurons, we were able to investigate their firing properties in wildtype mice that had been injected with the AAV-DJ-Cav3.2-mCherry virus. Voltage clamp experiments elicited typical T-type calcium currents from a holding potential of −90 mV (Fig. 6A). The current-voltage relationship of this voltage-sensitive calcium current plotted at low membrane voltages was consistent with a low threshold calcium channel^28^ in mCherry-positive neurons (n = 7, Fig. 6A) like in unidentified LII neurons encompassing Cav3.2-positive and negative neurons (Supplementary Fig. S4). In the current-clamp configuration, the peak amplitudes of the subthreshold rebounds were similar in Cav3.2-expressing neurons (7.6 ± 0.7 mV, n = 32, Fig. 6B) as compared to unidentified neurons (7.8 ± 0.6 mV, n = 57, Fig. 6B). Their time-to-peak was lengthened (unidentified neurons, 139 ± 8 ms, n = 57, and Cav3.2-expressing neurons, 193 ± 22 ms, n = 32; Mann-Whitney test, p < 0.001, Fig. 6C). Yet, T-type channels were strongly involved in these subthreshold properties, as rebounds were inhibited by puff applications of 1 µM TTA-A2 (n = 6, Wilcoxon paired test, p = 0.03, Fig. 6D). The other two types of subthreshold activities (hyperpolarizing and passive) were present in Cav3.2-expressing neurons (Fig. 6E), although their relative proportions were different as compared to unidentified neurons (Chi^2^ test, p = 0.006). The electrical activity was unique in Cav3.2-expressing, rebound-exhibiting neurons. Notably, the latency of an action potential (Fig. 6F), elicited upon step-depolarization, was shorter in rebound-exhibiting neurons (58.6 ± 7.4 ms, n = 31) as compared to hyperpolarizing- (168 ± 20 ms, n = 29) and passive-type of neurons (244 ± 47 ms, n = 31). Accordingly, the action potential threshold (Fig. 6G) was significantly lower in rebound-exhibiting neurons (−38.3 ± 1.1 mV) as compared to hyperpolarizing- (−32.6 ± 0.7 mV) and passive-type of neurons (−32.2 ± 0.7 mV). The time-to-peak of this action potential was also different in rebound-exhibiting neurons (2.57 ± 0.3 ms) as compared to the others (respectively 1.82 ± 0.2 and 1.80 ± 0.2 ms, in hyperpolarizing- and passive-type of neurons, Fig. 6H). When examining the following action potentials, rebound-exhibiting neurons showed a pairing of the first two action potentials, like in the unidentified population (n = 26, Wilcoxon paired tests, Fig. 6I). This property was not present in the two other populations of Cav3.2-expressing neurons, where the delay of the first action potential was distinguishable from the other intervals (respectively n = 27 and 26, Wilcoxon paired tests, Fig. 6I). Cav3.2-expressing neurons displayed the various firing patterns, as seen in unidentified LII neurons (Fig. 6J, and Supplementary Tables 5 and 6) and there was no all-or-none association between a subthreshold activity and a firing pattern, although rebound-exhibiting neurons tended to harbor transient firing behaviors, while action potentials were more likely delayed in hyperpolarizing-neurons (Fig. 6K). Collectively, these data suggest that Cav3.2-expressing neurons were not enriched with T-type calcium currents and T-type dependent firing patterns, as compared to unidentified LII neurons. This might be explained by a significant expression of Cav3.1 and Cav3.3 channels in Cav3.2-negative DH neurons^27^. Nevertheless, Cav3.2 seemed tightly involved in the subthreshold and suprathreshold properties of Cav3.2-expressing neurons.

### Deletion of Cav3.2 in the LII neurons of the spinal cord

**Figure 7:**
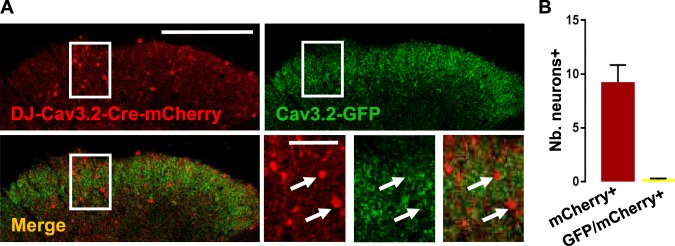
Selective deletion of Cav3.2 in the spinal cord of Cav3.2-GFP mice. (**A**) DH of the spinal cord slice from an adult Cav3.2-GFP mouse injected with a AAV-DJ-Cav3.2-Cre-mCherry and processed for immunodetections of GFP (green) and mCherry (red). mCherry-positive neurons lacked GFP expression as pointed out by the arrows in the expanded views of the box showed on the widest images. (**B**) Numbers of neurons expressing either mCherry alone or both GFP and mCherry per slice. Bars are the means and lines are the SEM of counts in 2 adult mice. Scale bars: **A** (200 µm), (50 µm).

Not surprisingly, Cav3.1 and Cav3.3 mRNAs could be detected in Cav3.2-expressing neurons (Supplementary Fig. S7). Like in primary afferent neurons^14,17,18^, ablation of Cav3.2 expression was therefore necessary for understanding its specific roles in the adult lumbar spinal cord. A virus encoding Cre-recombinase-IRES-mCherry under the control of Cav3.2 promoter was injected into the spinal cords of Cav3.2^GFP-Flox^ KI mice. As shown in Fig. 7A, the expression of Cav3.2-GFP was eliminated in Cre-recombinase- and mCherry-expressing neurons, and none of the mCherry-positive neurons were GFP-positive (Fig. 7B). Some cell bodies were GFP-positive and did not express mCherry (Fig. 7C) as expected in neurons that did not take up, nor express the AAV.

**Figure 8:**
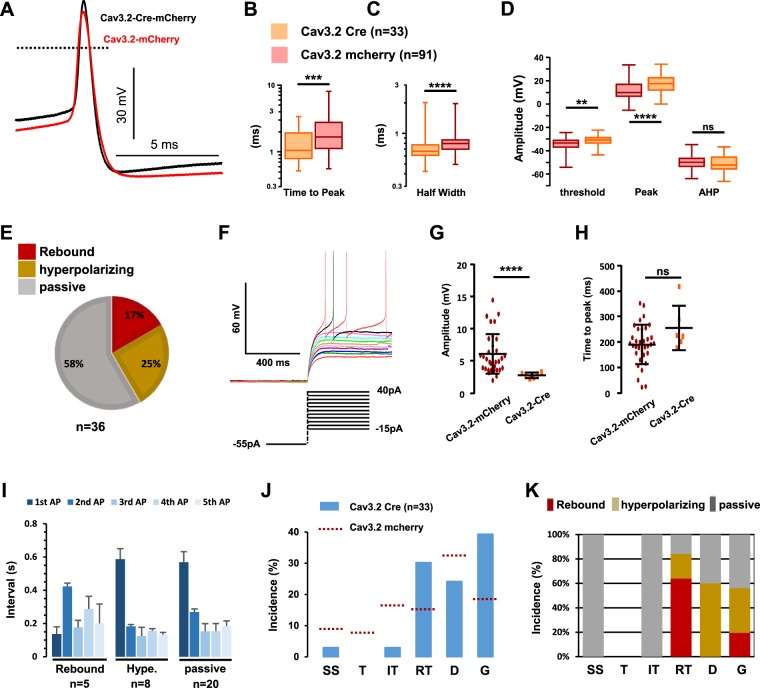
Electrophysiological properties of LII neurons lacking Cav3.2. (**A**) Raw traces of action potentials recorded in a Cav3.2-expressing neuron (red line) and a Cav3.2-deleted neuron (black line). (**B**–**D**) Box and whiskers representations of action potential’s time-to-peak (**B**), half width (**C**), and amplitudes (**D**) in mCherry-expressing neurons from either AAV-DJ-Cav3.2-mCherry injected mice (red) or AAV-DJ-Cav3.2-Cre-mCherry injected Cav3.2^GFP-Flox^ KI mice (orange). Significance ****p < 0.001; ***p < 0.005; **p < 0.01, using a Mann Whitney test. (**E**) Proportions of the subthreshold properties of Cav3.2-deleted neurons. (**F**) Voltage traces of subthreshold and supra-threshold properties of a Cav3.2-deleted LII neuron with rebound-type depolarisation. (**G**,**H**) Amplitudes (**G**) and time-to-peak (**H**) of the subthreshold rebounds in Cav3.2-positive neurons (red dots, n = 34) and Cav3.2-deleted neurons (orange dots, n = 5). Lines are mean and SEM. (**I**) Mean intervals showed no organization of the firing in Cav3.2-deleted neurons. Bars are the means and lines are the SEM Significances are indicated as: ****p < 0.0001; ***p < 0.001; **p < 0.01; *p < 0.05, using a Wilcoxon paired test. (**J**) Firing patterns in Cav3.2-deleted LII neurons from AAV-DJ-Cav3.2-Cre-mCherry injected Cav3.2^GFP-Flox^ KI mice, recorded in the current clamp mode of the whole-cell patch clamp technique. The dotted lines show these proportions in Cav3.2-expressing neurons (from Fig. 6J). (**K**) Relationships between firing patterns and subthreshold behaviors in Cav3.2-deleted LII neurons (n = 36). (**J**,**K**) SS (Single spiking), T (Transient), IT (Irregular Tonic), RT (Regular Tonic), D (Delayed) and G (Gap), see Fig. S3 for explanations.

The properties of mCherry-expressing neurons, that had lost the expression of Cav3.2-GFP, were then examined. Cav3.2 ablation did not change either the rheobase of the neurons (52 ± 2pA, n = 91, and 70 ± 10pA, n = 33, with and without Cav3.2, respectively). The kinetics of the action potentials were modified by the deletion of Cav3.2 (Fig. 8A–E). In the typical examples of Fig. 8A, the action potential of a Cav3.2-deleted neuron was more ample and faster than in a Cav3.2-expressing neuron. On average, half-width and time-to-peak were smaller (Fig. 8B,C) in Cav3.2-deleted neurons. In addition, both the threshold potential (Fig. 8D) and the peak potential (Fig. 8D) were shifted in Cav3.2-deleted neurons, whereas the after-hyperpolarization potential was not significantly increased (Fig. 8D). Cav3.2 was not only important for suprathreshold but also subthreshold properties since: (1) the proportions of rebound-exhibiting neurons and of hyperpolarizing neurons were decreased in Cav3.2-ablated neurons (Fig. 8E), as compared to Cav3.2-expressing neurons (see Fig. 6E) at the expense of passive-type of behavior (Chi^2^ test, p < 0.0001), and (2) as shown in the example of Fig. 8F, the remaining rebounds were barely detectable in Cav3.2-ablated neurons (3.0 ± 0.4 mV, n = 6, Fig. 8G) as compared to Cav3.2-expressing neurons (Fig. 8G, same data as in Fig. 6B), without alterations of their kinetics (Fig. 8H).

Cav3.2 ablation also impaired the overall firing of LII neurons. The profile of the firing of the first five action potentials was selectively modified in rebound-exhibiting neurons, as it did not show the pairing effect at the onset of activity (Fig. 8I), with the interval of the second action potential being variable from cell to cell. Yet, the latency of the first action potential was still shorter in rebound exhibiting neurons as compared to the two other populations (Fig. 8I). The firing patterns were also modified in Cav3.2-ablated neurons. The number of neurons exhibiting higher activities at the beginning of the pulse was diminished, as single spiking, transient and irregular tonic patterns were greatly down-sized in the distribution (n = 34, Fig. 8J). Thus, regular tonic and delayed patterns dominated this distribution. Finally, some relationships between subthreshold and suprathreshold properties were more apparent: most rebound-exhibiting neurons fired with a regular tonic pattern, and all hyperpolarizing neurons had delayed electrical activity, while passive neurons distributed in many classes (Fig. 8K). Note that the gap firing mode was equally represented in the three classes of neurons. Altogether, these results suggest a critical role of Cav3.2 channels in the subthreshold and suprathreshold properties of Cav3.2-expressing neurons of the LII of the spinal cord.

### Deletion of Cav3.1 in the LII neurons of the spinal cord

The phenotype of Cav3.2-ablated LII neurons might not be selective since some of them can also express Cav3.1 and Cav3.3 (see Supplementary Fig. S7), and T-type channels might reciprocally potentiate each other’s activity. This question was examined in Cav3.2-expressing LII neurons of Cav3.1 knockout mice^51^.

**Figure 9:**
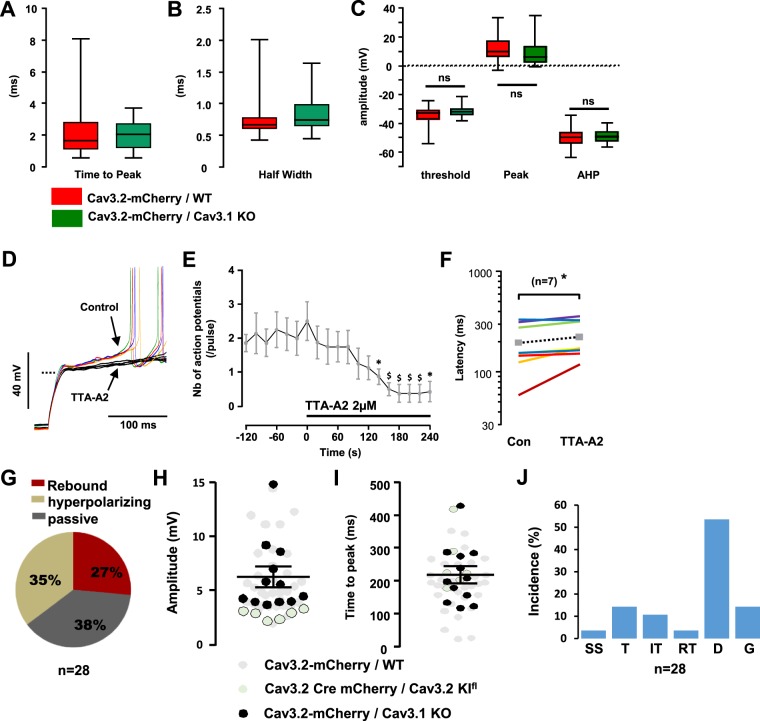
Electrophysiological properties of Cav3.2-expressing LII neurons lacking Cav3.1. (**A**–**C**) Box and whiskers representations of action potential’s time-to-peak (**A**), half width (**B**), and amplitudes (**C**) in mCherry-expressing neurons from either AAV-DJ-Cav3.2-mCherry injected wild-type mice (red, from Fig.) or AAV-DJ-Cav3.2-mCherry injected Cav3.1 knockout mice (green). In (**B** and **C**)**:** ^$^p < 0.01; ^*^p < 0.05, using a Wilcoxon paired test. (**D**) Raw voltage traces in a mCherry positive neuron, in the absence (colored lines) and presence of 2 µM TTA-A2 (black lines), recorded in a Cav3.1 knockout mouse injected with the AAV-DJ-Cav3.2-mCherry. Steps were elicited from ~−100 mV; the dotted line indicates −60 mV. (**E**) Mean number of action potentials triggered during a 200ms-long pulse (as shown in (**D**)), in the absence and presence of 2 µM TTA-A2 (as indicated by the solid line), in 7 Cav3.2-positive/Cav3.1-negative neurons. Symbols are the means and lines are the SEM. (**F**) Mean latency of the first action potential (same experiments as in **E**) triggered in the absence and presence of 2 µM TTA-A2 (as shown by the lines), in 7 Cav3.2-positive/Cav3.1-negative neurons. Symbols are the means. (**G**) Proportions of the subthreshold properties of Cav3.2-positive/Cav3.1-negative neurons. (**H**,**I**) Amplitudes (**H**) and time-to-peak (**I**) of the suprathreshold rebounds in Cav3.2-positive/Cav3.1-negative LII neurons (black dots, n = 12). Similar results from Cav3.2-expressing neurons (grey dots, from Fig.) and from Cav3.2-deleted neurons (white dots, from Fig.) are shown for a comparison. Lines are mean and SEM. (**J**) Firing patterns in Cav3.2-positive/Cav3.1-negative LII neurons, recorded in the current clamp mode of the whole-cell patch clamp technique. SS (Single spiking), T (Transient), IT (Irregular Tonic), RT (Regular Tonic), D (Delayed) and G (Gap), see Fig. S3 for explanations.

The absence of Cav3.1 in Cav3.2-expressing neurons did not change the properties of the action potentials in LII of the spinal cord (Fig. 9A–C), in marked contrast with the deletion of Cav3.2 described above. In addition, action potential firing was strongly dependent upon the activity of T-type channels, as shown in the experiment of Fig. 9D where superfusion with TTA-A2 (2 µM) abolished firing induced by a current injection from −90 mV. On average, TTA-A2 superfusion quickly blunted the excitability of Cav3.2-expressing neurons lacking Cav3.1 (n = 8, Fig. 9E) and significantly increased the first action potential latency in trials were firing was observed (from 195 ± 35 to 224 ± 34 ms, n = 7, p = 0.02, Wilcoxon paired test, (Fig. 9F). Furthermore, the distribution of the subthreshold activities was almost superimposable in Cav3.2-expressing neurons (see Fig. 6E) and in Cav3.2-expressing neurons lacking Cav3.1 (Fig. 9G). Accordingly, the maximal amplitudes (Fig. 9H) and the times-to-peak (Fig. 9I) of the rebounds induced by step-depolarization were similarly distributed in the 3 to 15 mV range in Cav3.2-expressing with or without Cav3.1. The distribution of the firing patterns was not greatly altered in Cav3.1 knockout mice since 1) immediate firing (single spiking, transient and irregular tonic, Fig. 9J) amounted to ~29%, and was close to the 32% seen in Cav3.2-expressing neurons (see Fig. 6J) and 2) delayed and gap firing patterns were as represented in Cav3.2-expressing LII neurons with or without Cav3.1 (respectively, 50% and 68%). Yet, the downsizing of the regular tonic firing pattern from 15% to 4% of the Cav3.2-expressing neurons (compare Figs 6J with 9J) was opposite to the effect of Cav3.2 ablation (30%, in Fig. 8J). Altogether, these data show that spinal deletion of Cav3.2 has profound effects on the excitability of most classes of LII neurons while Cav3.1 deletion does not recapitulate these changes.

## Discussion

The Cav3.2^GFP-Flox^ KI mouse was instrumental in deciphering the role of Cav3.2 in primary afferent neurons during chronic pain^14^. Here, this mouse model was used as a genetic tool for identifying Cav3.2 expressing neurons and for ablating them in LII of the spinal cord where Cav3.2 was found to play an important, and specific role in the subthreshold and suprathreshold properties.

### Synaptic transmissions

Primary afferent fibers, including the C-LTMR and the Aδ-LTMR, express Cav3.2 and project to the DH of the spinal cord^14,15,52,53^. GFP detection in the DH of the Cav3.2^GFP-Flox^ KI mouse suggests that Cav3.2 has a presynaptic role^14^. Indeed, projection neurons of the superficial lamina I are modulated by primary afferent fibers in a T-type dependent manner^6,8^, that is impaired in Cav3.2 knockout mice^10^. In contrast, mEPSCs were not significantly modified by T-type channel inhibitors in LII neurons. There is a gap between these sets of data: the small population of projection neurons, which is rather homogenous^54^, was examined after retrograde labeling^6,8,10^, while the current data were obtained in unidentified LII neurons, that is, a heterogeneous population where some, rather than all, excitatory inputs might be Cav3.2-dependent^5,9,11^. The role of Cav3.2 in glutamatergic transmission will have to be examined in targeted, well-defined, LII neurons, possibly in combination with cell-type specific activation of primary afferent neurons. Surprisingly, TTA-A2 slowed mIPSCs in LII neurons, and the selective inhibition of mIPSC frequency was suggestive of a presynaptic site of action of the T-type channel inhibitor. Note that the use of TTA-A2 at concentrations in the micromolar range precludes this compound from blocking high-voltage activated calcium channels in multicellular preparations^55,56^. Altogether, the distribution of Cav3.2 at inhibitory synapses of LII neurons appeared more homogenous than at excitatory synapses. It will be interesting to investigate if these synapses arise from local interneurons and/or from descending inhibitory pathways. Note that T-type channel inhibition does not seem as tightly involved in the GABAergic neurotransmission in other preparations^9,10^. Some methodological differences between these and our study might be tested in the future (namely, animal species and age, recording conditions including pharmacology).

### Action potential properties

As suggested by the dense Cav3.2 labelling of neuronal cell bodies in LII, the intrinsic properties of LII neurons involved a contribution from Cav3.2. First, the early steps of the action potential (time-to-peak, threshold and peak amplitudes) were modified by the ablation of Cav3.2, as expected for a protein involved in subthreshold properties. Cav3.2 ablation not only diminished the proportion of rebound-exhibiting neurons and the amplitude (if any) of this rebound, since some suprathreshold properties of the hyperpolarizing and the passive neurons were also altered in Cav3.2 deleted LII neurons (Supplementary Table 7). Accordingly, the proportion of LII neurons with T-type calcium currents (~75%) is larger than the rebound-exhibiting population (~30%). In apparent contrast, Cav3.2 ablation modified neuronal excitability without changing action potential threshold in Aδ-LTMR cell bodies^57^. Keeping in mind that Cav3.2 can be found at the axon initial segment, others have suggested that its contribution to neuronal spiking might have profound, albeit non-conventional, effects^58,59^. For a comparison, the deletion of Cav3.1 did not mimic these changes, suggesting that Cav3.1 might be relevant in other compartments of the LII neurons. Alternately, Cav3.1 might not have a homogenous role in Cav3.2-expressing LII neurons. Second, Cav3.2 ablation shortened the half-width of the action potential in LII neurons, and this occurred with a significant change of the AHP in rebound-exhibiting neurons only (Supplementary Table 7). T-type channel inhibition decreases the amplitude of the AHP in other neurons as well^6,60^. Third, the mean action potential latency was not significantly increased in Cav3.2-ablated LII neurons as compared to Cav3.2-expressing neurons despite the subthreshold role of Cav3.2. The mean action potential latency was also similar in Aδ-LTMR cell bodies of either Cav3.2-deleted mice or wild-type mice^57^. Altogether these data establish that Cav3.2 has unique roles in the LII neurons, which should be examined further in the multiple subpopulations found by Zeisel *et al*.^61^.

### Firing patterns and spike coding

The firing patterns of neurons are heterogeneous in LII^12,29,30,32,34^, and are not less heterogeneous in Cav3.2-expressing neurons. Because of their inactivation properties, T-type calcium channels are first viewed as supporting transient activities^50,62^. Cav3.2 ablation indeed lowered the population of LII neurons with immediate activity, encompassing single-spiking, transient, and irregular tonic patterns. Cav3.2-ablated neurons exhibited mostly delayed and gap patterns. Being driven by transient potassium channels, their firing delays were expected to be exacerbated, if anything, in Cav3.2-ablated neurons^32,63^. The transition from transient behaviors to more regular tonic patterns might be related to the simultaneous decrease in the proportion of rebound-exhibiting neurons and in the decrease in the amplitude of the rebound in the remaining neurons. T-type calcium channels also contribute to tonic firing when they generate a tonic “window” depolarisation^12,13,64,65^. In thalamic neurons, where peak T-type currents are in the nA range, the tonic contribution of T-type channels is ~2mV^36^. Since T-type currents were much smaller (10–100 pA range) in LII neurons, it was not surprising that the proportions of regular firing patterns (regular tonic + delayed) and of irregular patterns (irregular tonic + gap) were similar in Cav3.2-expressing neurons and Cav3.2-ablated neurons. A putative tonic contribution of Cav3.2 to the resting potential might better be examined in combination with varying synaptic strengths^64^.

Cav3.2 likely plays a role in the coincidence detector function of LII, as suggested by the result of Cav3.2-ablation. First, spike initiation being influenced by T-type channels, variations or regulations of Cav3.2 activity will control the precision of the temporal summation of synaptic inputs^64^. Second, delayed excitation (a rebound of the membrane potential triggered by inhibitory transmission) is strongly dependent upon T-type channels activity^31,66^, and might largely depend upon Cav3.2 in LII neurons (this study). Third, the downstream transmission of the sensory signal depends on neuronal firing patterns. Single spiking patterns, blunted in Cav3.2-ablated neurons, convey spike time precision and the opportunity in pairing synaptic inputs on postsynaptic neurons^64,67^. Altogether, Cav3.2 ablation perturbed millisecond coding properties of LII neurons, required for temporal neural codes^68^.

The action potential pairing, eliminated by Cav3.2 ablation in LII neurons, shared some, but not all, properties with other neurons. Indeed, delayed doublet spiking also occurs in sensory neurons of an electric fish, but this is a TTX-sensitive event which terminates bursts^69^. Doublet spiking is followed by an AHP which silences electrical activity in motoneurons, and this might facilitate force generation^70^. In mature granule neurons of the dentate gyrus, Cav3.2 supports action potential pairing within tenth of ms, while T-type channel antagonists or Cav3.2 genetic ablation lengthen this interval to no more than 75 ms^71^. In the cerebellum, long term depression at the parallel fiber – Purkinje neuron excitatory synapse is better induced by a fast pairing of parallel fiber stimulation (interval 60 ms)^72^. These doublets, whose functions are identified, are much faster than those elicited by T-type dependent rebounds in LII neurons and fall into the 50–200 ms range. Yet, these kinetics fit with the hypothesis of Cav3.2 participating in paired pulse facilitation/depression at inhibitory synapses in the DH of the spinal cord^67^. In support for this hypothesis, TTA-A2 blunted the spontaneous inhibitory currents in LII neurons in a manner consistent with a presynaptic mode of action. However, the other T-type channels likely participate in synaptic inhibition as well, because Cav3.2 was not common in Pax2-expressing neurons. Recording from identified synapses, which is becoming feasible with the classification of neuronal subpopulations^61^, will be required in resolving these issues.

### Functional specificity

Some Cav3.2-expressing neurons expressed Cav3.1 or Cav3.3 in the spinal cord. Despite similar biochemical and electrophysiological characteristics, T-type channels can be discriminated from each other^73,74^. In addition, their subcellular localizations are not always overlapping^75^. In LII neurons, Cav3.1 and Cav3.2 were not redundant since the subthreshold and suprathreshold properties of Cav3.2-expressing neurons were similar in Cav3.1 knockout mice and in wildtype mice. A putative cooperativity between Cav3.1 and Cav3.2 did not take place in LII neurons either, as Cav3.1 deletion had minimal effects (if any) on excitability while Cav3.2 deletion seemed to eliminate most if not all signatures of T-type calcium channels. Future sets of experiments, including afferent synaptic activation for instance, might challenge this conclusion. In addition, the ~3.0 ± 0.4 mV mean rebound remaining in Cav3.2-ablated neurons (see Fig. 8G) might not be negligible, and its putative mechanism remains to be clarified (the residual redound in the presence of TTA-A2 being in the ~1 mV range). The unique role of Cav3.2 was also suggested by the unusual proportion of passive subthreshold behaviors in Cav3.2-deleted LII neurons. Another comparison, between the distributions of subthreshold properties in unidentified neurons and Cav3.2-expressing neurons suggest an electrogenic role of either Cav3.1 and/or Cav3.3 in Cav3.2-negative neurons notably because rebounds were more common in unidentified neurons. The kinetics of the rebounds, shorter in unidentified neurons, again suggested distinct properties between Cav3.2-positive and Cav3.2-negative LII neurons. Future experiments will be needed in deciphering the roles of Cav3.1 and Cav3.3 in the spinal cord, keeping in mind that inactivation of their genes does not induce analgesia, so far^16,17^.

Our immunostaining and the *in situ* hydridization experiments clarified the heterogeneity of the Cav3.2-expressing neuronal population. So far, Cav3.2 colocalized mainly with excitatory neurons in the general population as well as in each of the subpopulations examined, but the PV neurons. Importantly, Cav3.2 is present in almost each PKC_ϒ_ interneuron, and these neurons are on the pathway of non-nociceptive information to the projection neurons of lamina I^21–26^. Interestingly, a large population of CR neurons (41%) is also Cav3.2-positive, and this proportion is somewhat higher than suggested by the patch-clamp recordings of Smith *et al*.^32^. Unlike PKC_ϒ_ interneurons, CR neurons seem to be involved in inflammatory pain and unrelated to the onset and development of mechanical allodynia^76^. The role(s) of Cav3.2 in CR is/are as yet unknown, and the development of new transgenic mice models and/or new AAV will be useful in examining these functions. In contrast to these Cav3.2-positive neurons that allow the propagation of the allodynic signal to the projection neuron, the PV neurons which gates this pathway^26^ did not express Cav3.2. Although the understanding of T-type channel functions within the spinal cord still remains to be fully elucidated, they are likely to tune the excitability of some spinal neuronal networks, the pharmacological modulation of which might be of therapeutic importance.

## Supplementary information

Supplementary figures

Supplementary Tables
