# Supplementary material for: Cav3.2 T-type calcium channels shape electrical firing in mouse Lamina II neurons
Source: Sci Rep. 2019 Feb 28;9:3112. doi: 10.1038/s41598-019-39703-3 (PMC6395820; doi:10.1038/s41598-019-39703-3)
Supplement: Supplementary file 1 — Supplementary figures [file 41598_2019_39703_MOESM1_ESM.pdf]

SUPPLEMENTARY FIGURES

# Cav3.2 T-type calcium channels shape electrical firing in mouse Lamina II neurons

**Miriam Candelas [1,2,3,4], Ana Reynders [5], Margarita Arango-Lievano [2,3,4], Christoph Neumayer [1,2,3,4], Antoine Fruquière [1,2,3,4], Elsa Demes [1,2,3,4], Jawed Hamid [6], Céline Lemmers [1,2,3,7], Claire Bernat [1,2,3,7], Arnaud Monteil [1,2,3,4,7], Vincent Compan [1,2,3,4], Sophie Laffray [1,2,3,4], Perrine Inquimbert [8], Yves Le Feuvre [9], Gerald W. Zamponi [6], Aziz Moqrich [5], Emmanuel Bourinet [1,2,3,4*], Pierre-François Méry [1,2,3,4 *]**

***ACGC***GTTCAAGGCTCAAGGCTCTTCCTGATCCCTCTCCTGGGTGGCTAGGTCCTTAATTGCTCTTTGA

***MLUI***

GGACCTTAATTATGGGCCGCAGCCACCCACTGGTAAGTAGGAGCATTAAAATAGAGTAAATGTTTTTC

TTTTCTTGAAAAGTTCACATATACAAGACAAGGTGCTGTTGGGCCCAGGGGCCTGCCCTGCCTGGACC

GACAGGCAGAGGACATGGTTCTTGTTCTCCACCTCCTTCCCCAGGCTGTGCCCTTCCCAGGCTCAGTG

GTGAAATCCTCTTAGAATCCCAGAACCACATATGGGAAACTCCACTTCTCTGCAGCTCACCCTGCCAG

GGCTCCATCCAGGTCTGAGGTTGCTATGTTGCTCTGGCCCTCCAAAGAGCCCAGCTACCCCAGGGCAG

AGGGAGGCTGCTCTTTGTCTAGGACAGGAATGGTTTTCCCCAATAACAGGGCCTGAAGGTGAAAGAGC

CCCTTGTTGGTTGAAATTTAAGTGGATGTACACCTAAGAGCTGGAATTTTATGGCCCTAGTGTGGTAA

TTGTGAGAAAGTAAAAAAATCCCTGCATACCTCCAGCGGGAGAGGGGGGGAACAGCCCCTCTGCTCAT

GCCTTCATGGGACAAGGGTCATGCAACCACTGGAGTATGAAAGGAGATTCAGCGACATGCTCAGGATA

TACCCTCTGTGTGCCTTGGCGGGTGCTGGGAGGCCCCGCTCGGCTTGTACTCCTTCTCGAGGTTCTTC

CAAGAGCCCCAGTCCCCAGGAGAAGGGGGAGGGGGATGGGCACCGGCAAGTTTGCGGAAACTGGGAGG

AGTGTGGGGGAGCTGCGTACCAGAGTACCCGGACTGCCCCAGGACAATTGGTGTCGCGTCGCGCAACG

CGCCCCACGTCGCCCACGCGCCCCAAAGACACTGGCGCCCCGGCACACCCAAGACACACGCGGTGGGG

GAAGGGCACGCGCCTCGCGGGCATGGTCACGGCGTGCCTCCCACGCCTGCTGAGCCCCGCCCCCTAGC

CGACCCTAGCAGTGAAGAGAAGGGGCGGCGCGCGAGAAAAGGAGGGGGTCCCCGCCGGCTCCGCGCCC

CCGCAGTGGGGGTGGGGAAGGACGTTGGCGCCGCTTCCCCCGCCCCTCGTCCAAGCATCCCTGCGAGC

GCCCCCTGCTGCCGTGCTCTCCCGCGAGACAGTATGGGGCGGGAGGAGAGGTCGGGGAGGGGGGCGCG

GACCGGGTTCGCCCAGTCCTGGACTTGGGGCTGGCGGCGTACCGGCACGGAGAAACACGGACCAAGGG

ATGTTGTGGGATAGCTTACGTCTGGGTGGGGGCGGGCCGGGCTGATGACCCCCGCCCACTCTCCGTCC

CTATCTCGGTCCCTGAGGGTCGCCCCCTGCCCGCCCCGGCCGCGTTGGCCCCGCCCCATGGGCCGCCC

CCGCCGCCCCGCCTCCCCGCCACTGCTCACTCCGAAGTTT***GGATCC***

***BAMHI***

**Supplementary Figure S1. Minimal mouse Cav3.2 promotor sequence of the AAV constructs.** Core promoter is presented in bold capital letters flanked with extra MluI and BamHI sites (in italics) used for cloning.

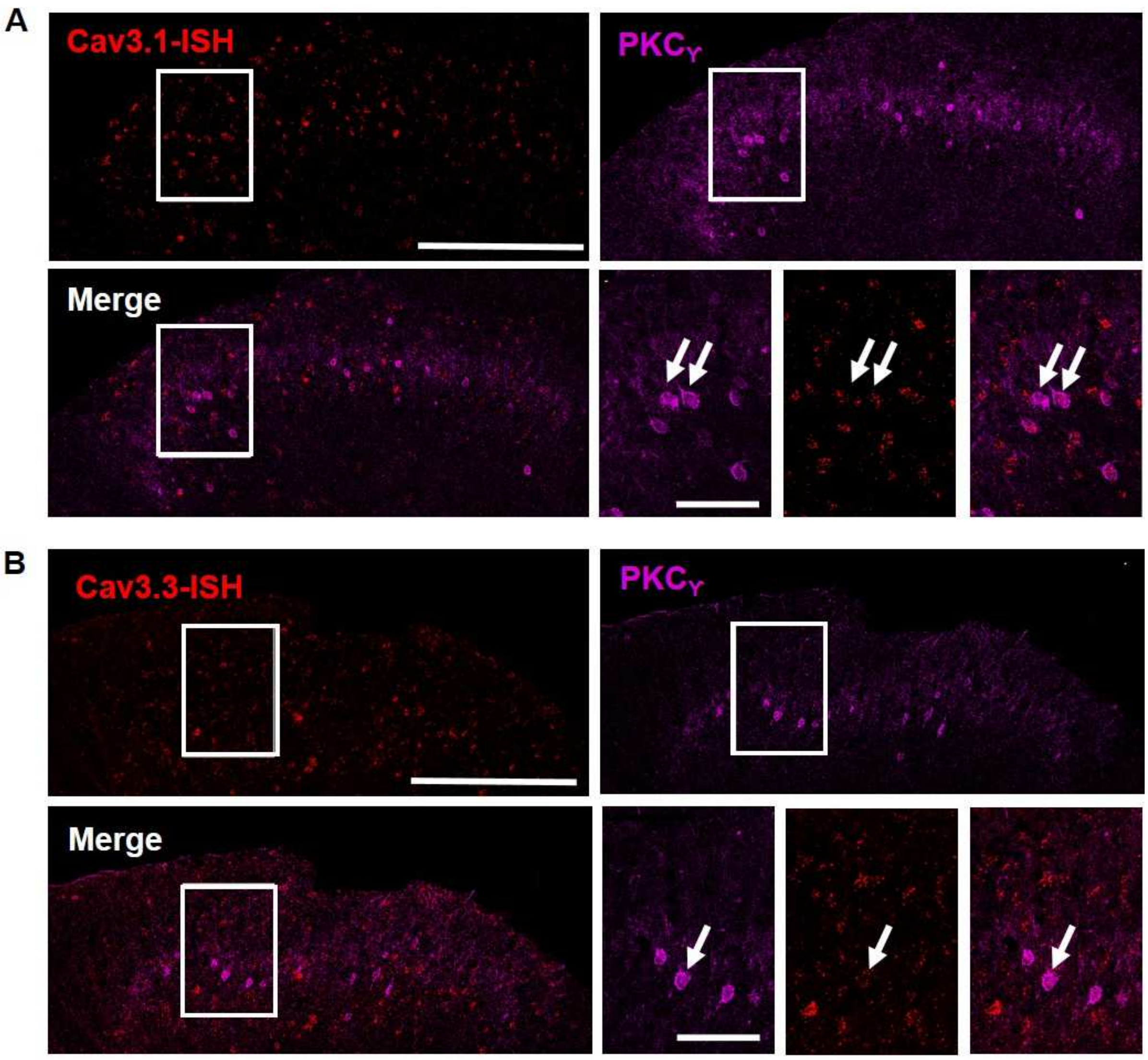


**Supplementary Figure S2. PKCϒ neurons express Cav3.1 and Cav3.3 in the dorsal horn of the spinal cord of adult mice. (A)** Sections of spinal cord at the lumbar level demonstrating overlaps in the expression of Cav3.1 mRNA (red), by means of *in situ* hydridization (see Table 2 for probe sequence), and of PKCϒ immunoreactivity (blue) detected with a rabbit anti-PKCϒ.and amplified with a donkey anti-rabbit A647 antibody. **(B)** Same as in **(A)** except that the Cav3.3 (red) was revealed by *in situ* hybridization. Arrows in **A** and **B** show coexpressions of markers. Scale bars: **A, B** (long=200µm, and short=50µm).

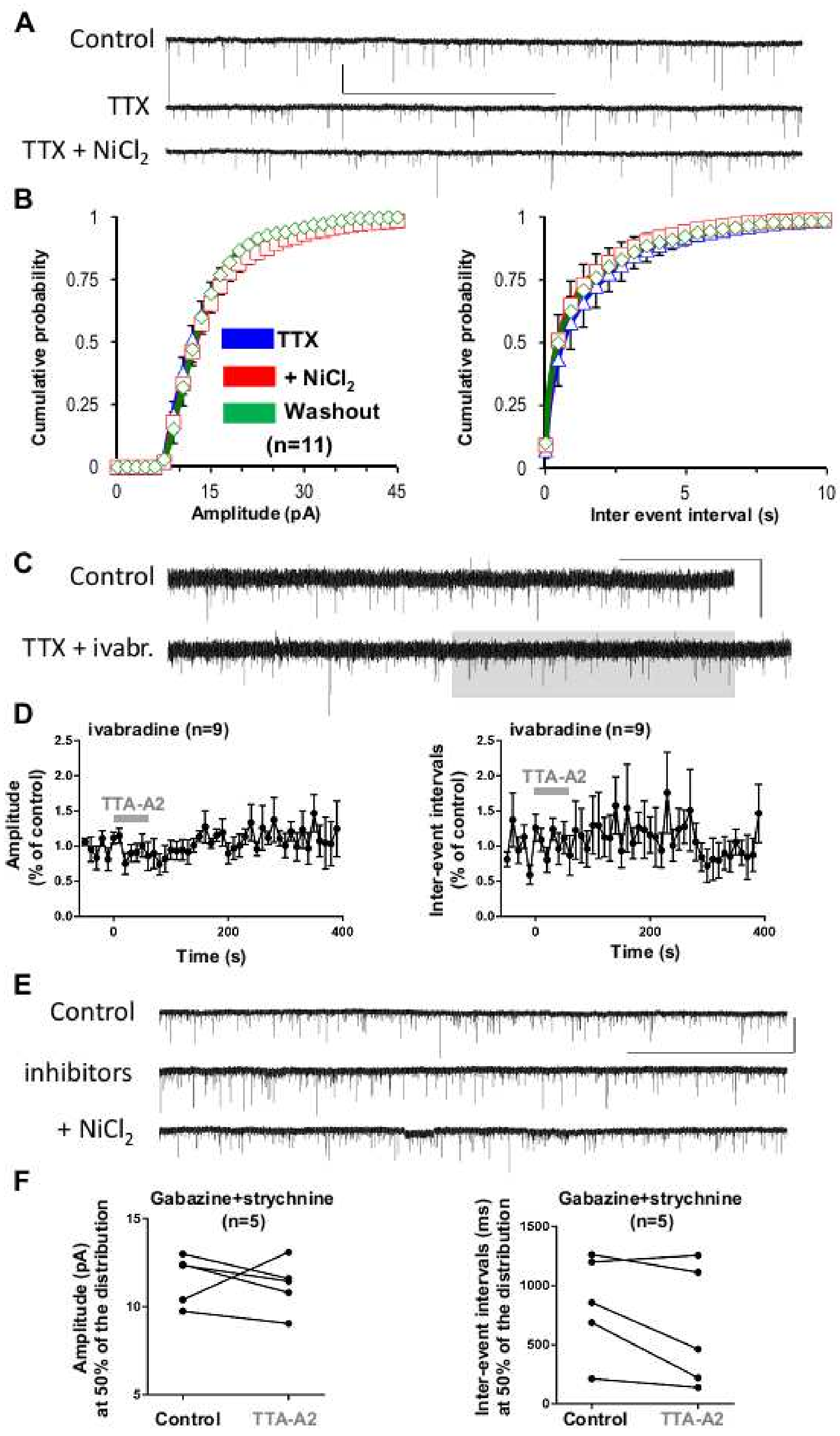


**Supplementary Figure S3. T-type calcium channels do not control the mean excitatory neurotransmission of LII neurons in the spinal cord of adult mice.** (**A**) Excitatory synaptic currents recorded successively under control conditions, in the presence of TTX 500 nM and in the presence of TTX + $NiCl_2$ 100 µM in a LII neuron held at -70 mV. Note that TTX diminished the synaptic activity, which was not altered further by $NiCl_2$. Scale bars: 40 pA, 1 min. (**B**) Mean cumulative distributions of the amplitudes (**left panel**) and the inter-event intervals (**right panel**) of mEPSC in the

absence (TTX 500 nM) or during the superfusion with 100µM $NiCl_2$ (n=11). **. (C)** Excitatory synaptic currents recorded in a LII neuron held at -70 mV, under control conditions, and in the presence of TTX 500 nM + ivabradine 1 µM, a HCN inhibitor (see Huang et al., 2011), without or during ejection of 1 µM TTA-A2 (shaded area). Scale bars: 40 pA, 30 s. **(D)** Mean amplitudes **(left panel)** and inter-event intervals (**right panel**) of mEPSC before, during and after a 1 min-long ejection of 1µM TTA-A2 (start at time 0) in the continuing presence of ivabradine 1µM (n=9). Data are shown as series of 10s-long bins, normalized to the levels recorded before TTA-A2 ejection. **(E)** Excitatory synaptic currents recorded successively under control conditions, in the presence of TTX 500 nM + Gabazine 3 µM + strychnine 3 µM, without or with $NiCl_2$ 100 µM in a LII neuron held at -70 mV. Note that the inhibitors increased inter-event intervals, while $NiCl_2$ had a marginal effect on the synaptic activity. Scale bars: 30 pA, 40 s. **(F)** Midpoints of the cumulative distributions of the amplitudes **(left panel)** and the inter-event intervals (**right panel**) of mEPSC in the absence (control) and during the superfusion with TTA-A2 500nM in 5 separate experiments performed in the continuing presence of Gabazine 3µM and strychnine 3µM, respectively inhibitors of GABA-A and glycine receptors.

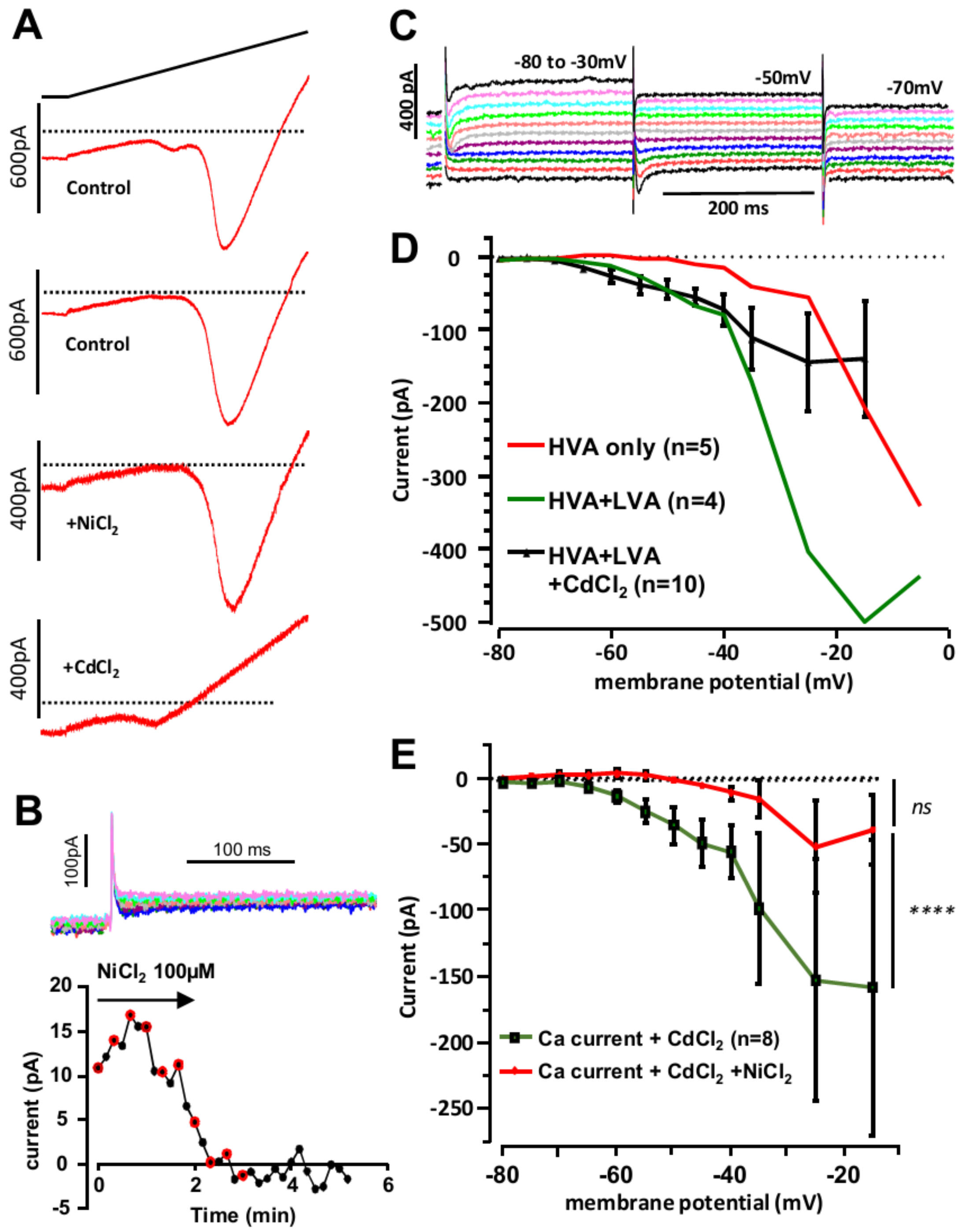


**Supplementary Figure S4. T-type currents of LII neurons in the spinal cord of adult mice. (A)** Voltage ramp relationships of four different LII neurons recorded under control conditions and in the presence of either 100µM $NiCl_2$ or 100µM $CdCl_2$. The dotted line shows the 0 pA level. The 200ms-ramp is indicated by the upward-going line. **(B)** Typical experiment in a LII neuron where the inward current recorded at -65 mV from a holding potential of -90 mV was inhibited by the superfusion with 100µM $NiCl_2$. The superimposed current traces are taken at the times indicated by the red symbols. **(C)** Family of current traces elicited by step-depolarisations (5mV increments) in a LII neuron from a holding potential of -90mV. Traces were shifted step-by-step to the top, for clarity. **(D)** Mean current-

voltage relationships of LII neurons (protocols as in **C**) recorded in the absence (colored lines) and presence (black line and symbols) of 100µM $CdCl_2$. Neurons without low-voltage activated inward currents (red line) are compared with neurons exhibiting both low- and high-voltage activated currents (green line, and black symbols) **(E)** same as in **D** except that the calcium current remaining in the presence of 100µM $CdCl_2$ was inhibited by 100µM $NiCl_2$. *ns*, non significant; ***, $p<0.005$, using a Wilcoxon paired test. **(A-E)** all recordings were performed in the presence of extracellular DNQX (20µM), APV (50µM), and Gabazine (3µM). In **D** and **E**, squares and vertical lines are for the means and the SEM. See Methods for additional information.

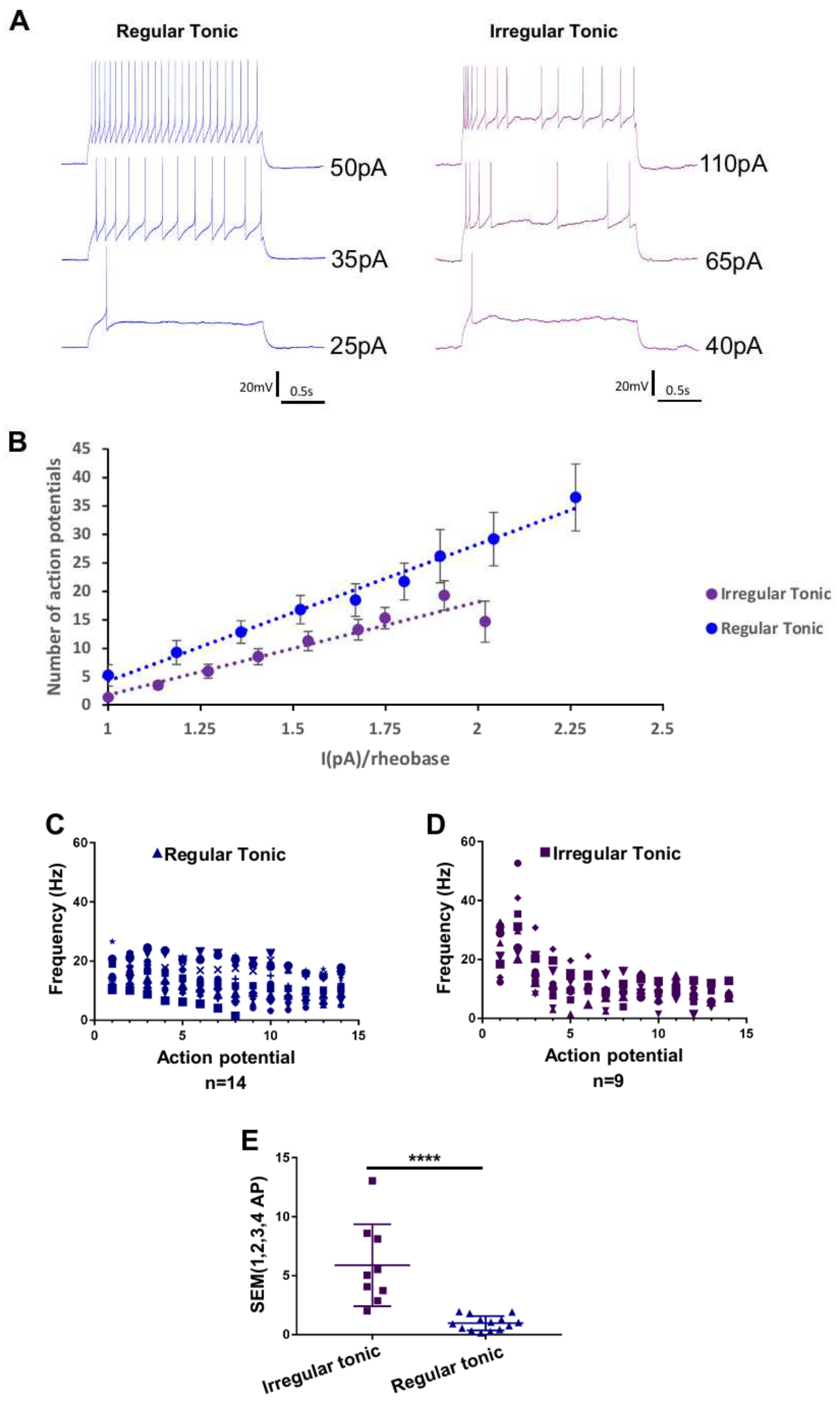


**Supplementary Figure S5. LII neurons displayed two types of tonic firing patterns in adult mice.** **(A)** Typical recordings of regular tonic and irregular tonic firing patterns at three current injection amplitudes. **(B)** Mean number of action potentials (elicited during a 2 sec-pulse, see Methods) as a function of the ratio (amplitude of the stimulatory current / rheobase) in tonic LII neurons. Note that 1) regular tonic neurons (n=10) are more active than irregular tonic neurons (n=14) and 2) the relationship is more likely linear (dotted line, $r^2$=0.98) in the regular tonic subpopulation as compared to the irregular tonic subpopulation (dotted line, $r^2$=0.91). **(C,D)** Instantaneous frequencies of the first 15 action potentials elicited by current injections in different neurons. **(E)** Calculation of the SEM of the frequency of the first 4 action potentials is sufficient to discriminate between regular and irregular tonic regime in LII neurons. Lines are means and SEM, and dots are the individual values. **** p<0.0001 using a Mann-Whitney test.

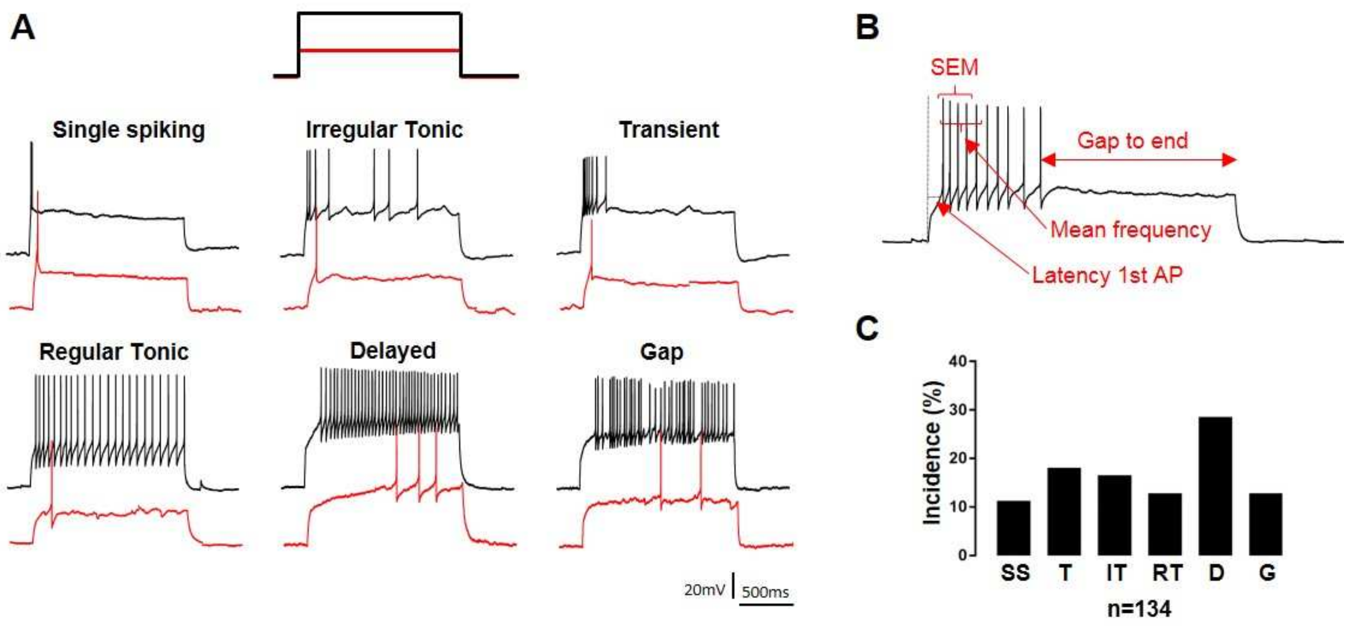


**Supplementary Figure S6. LII neurons displayed different types of discharge profiles. (A)** Individual traces of the firing patterns in LII neurons in adult mice. Current injections were performed after setting a resting potential at -70mV. Red traces are the activity at rheobase, and black traces are the activities at steady state regime. **(B)** Parameters allowing the classification of firing patterns of LII neurons. SEM (Standard error of the mean), AP (Action potential). **(C)** Graph showing the proportions of neurons expressing different profile discharges (n=134). SS (Single spiking: less than 2 action potentials per pulse), T (Transient: activity duration < 1.4s in a 2s-long pulse), IT (Irregular Tonic: tonic and having a SEM of the first 4 action potential intervals >2), RT (Regular Tonic: tonic and having a SEM of the first 4 action potential intervals <2), D (Delayed: the latency of the first action potential >95ms and a mean frequency of the first 5 actions potentials >8Hz) and G (Gap: the latency of the first action potential >95ms and a mean frequency of the first 5 action potentials <8Hz).

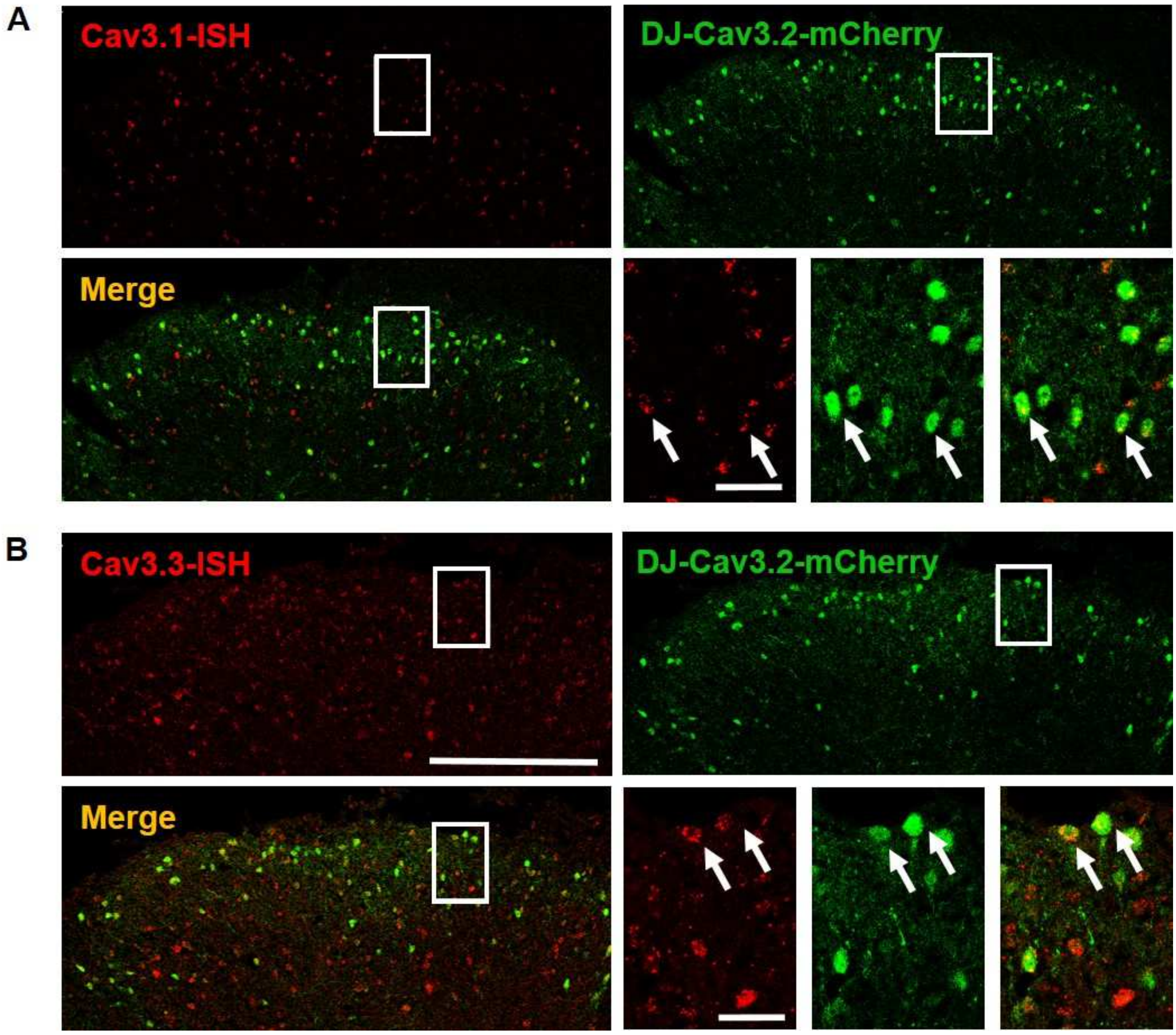


**Supplementary Figure S7. Cav3.2-mCherry neurons expressed Cav3.1 and Cav3.3. (A)** Colocalization of Cav3.1 mRNA (red, supplementary Table S2 online, for probe sequence) by *in situ* hybridization and mCherry (green) by immunodetection in wildtype mice injected with AAV-Cav3.2-mCherry. mCherry was detected with an anti-GFP antibody (supplementary Table S1 online). Arrows show coexpressions. **(B)** same as in **(A)** except that Cav3.3 mRNA was targeted by *in situ* hybridization. Data representative of 3 mice. Scale bars: **A,B** (200µm, 25µm).

References:

Huang, Z., R. Lujan, I. Kadurin, V. N. Uebele, J. J. Renger, A. C. Dolphin and M. M. Shah (2011). "Presynaptic HCN1 channels regulate Cav3.2 activity and neurotransmission at select cortical synapses." Nat Neurosci **14**(4): 478-486.
