# Supplementary material for: Cav3.2 T-type calcium channels shape electrical firing in mouse Lamina II neurons
Source: Sci Rep. 2019 Feb 28;9:3112. doi: 10.1038/s41598-019-39703-3 (PMC6395820; doi:10.1038/s41598-019-39703-3)
Supplement: Supplementary file 2 — Supplementary Tables [file 41598_2019_39703_MOESM2_ESM.pdf]

Supplementary Tables

Cav3.2 T-type calcium channels shape electrical firing in mouse Lamina II neurons

Miriam Candelas [1,2,3,4], Ana Reynders [5], Margarita Arango-Lievano [2,3,4], Christoph Neumayer [1,2,3,4], Antoine Fruquière [1,2,3,4], Elsa Demes [1,2,3,4], Jawed Hamid [6], Céline Lemmers [1,2,3,7], Claire Bernat [1,2,3,7], Arnaud Monteil [1,2,3,4,7], Vincent Compan [1,2,3,4], Sophie Laffray [1,2,3,4], Perrine Inquimbert [8], Yves Le Feuvre [9], Gerald W. Zamponi [6], Aziz Moqrich [5], Emmanuel Bourinet [1,2,3,4*], Pierre-François Méry [1,2,3,4 *]

**Supplementary Table 1 : antibodies for immunoflourescence experiments**			
**Antigen**	**Species**	**Dilution**	**Supplier**
**Primary antibodies**			
GFP	Chicken	1/250	Invitrogen
RFP-pAB	Rabbit	1/500	Medical & Biological Laboratories
Tlx3	Guinea pig	1/20000	Gift by Dr. Muller (Muller, Anlag et al. 2005)
Pax2	Rabbit	1/50	Invitrogen
NeuN	Rabbit	1/1000	Millipore
Calbindin D-28k	Rabit	1/1000	Swant
Calretinin	Mouse	1/1000	Swant
PKCϒ	Guinea pig	1/700	Frontier Institute
PKCϒ	Rabbit	1/1000	Santa Cruz Biotechnology
Parvalbumin	Rabbit	1/1000	Swant
nNOS	Sheep	1/3000	Gift by Dr. Prevot (Herbison, Simonian et al. 1996)
Tlx3	Rabbit	1/20000	Gift by Dr. Muller (Muller, Anlag et al. 2005)
NeuN	mouse	1/1000	Millipore
**Secondary antibodies**			
Anti chicken CF488	Donkey	1/500	Sigma
Anti guinea-pig A647	Goat	1/500	Invitrogen
Anti rabbit CF350	Donkey	1/500	Biotum
Anti rabbit Cy3	Goat	1/500	Jackson
Anti mouse A568	Goat	1/500	Invitrogen
Anti sheep A647	Donkey	1/500	Invitrogen
Anti rabbit Cy3	Goat	1/500	Jackson
Anti mouse A568	Goat	1/500	Invitrogen
Anti sheep A647	Donkey	1/500	Invitrogen

Supplementary Tables (continued)

**Supplementary Table 2: primer sequences used for in situ hybridization**		
Gene	Primer	Sequence (5’ → 3’)
Cacna1i	F1	AGCAGCCTGTCACTCACATC
	R1	GGTGTGAACTGTCCCCCAAA
	F2	AGTTCAGCAGTACCAGCAGC
	R2	**TAATACGACTCACTATAGGG**GTCCCCCAAAGTGTTCACGA
Cacna1g	F1	GCCAAGGACCCTCAAGAGAC
	R1	TGTAATACACTCACGGCCCC
	F2	AAGGACCCTCAAGAGACCAGA
	R2	**TAATACGACTCACTATAGGG**CAGACGCTTTCGCCTCAAAA
Cacna1h	F1	GGAAGGGCAGATAGATGACG
	R1	GTCTTATTGCATGGGCCAGT
	F2	CGGAGCCTGCTGAAAATATC
	R2	**TAATACGACTCACTATAGGG**GAAGGATGCCGAGTGATGATT

Bold text : T7 promoter sequence

Supplementary Tables (continued)

Supplementary Table 3. Neurons and mice used for statistical analysis in Figures.

Figure	Neuron type	Nb of neurons	Nb of mice
Figure 3C, 3D	Unidentified	12	8
Figure 3G, 3H	Unidentified	12	4
Figure 4B, 4C	Unidentified	124	56
Figure 4F	Unidentified	7	4
Figure 4H	Unidentified	101	56
Figure 6A	Cav3.2 mcherry	7	3
Figure 6B, 6C	Unidentified	57	27
	Cav3.2 mcherry	32	23
Figure 6D	Cav3.2 mcherry	6	3
Figure 6E-6H	Cav3.2 mcherry	119	40
Figure 6I-6K	Cav3.2 mcherry	91	40
Figure 8B-8D	Cav3.2 mcherry	91	40
	Cav3.2 Cre mcherry	33	11
Figure 8E	Cav3.2 Cre mcherry	36	11
Figure 8G, 8H	Cav3.2 mcherry	34	23
	Cav3.2 Cre mcherry	5	4
Figure 8I	Cav3.2 Cre mcherry	32	11
Figure 8J, 8K	Cav3.2 Cre mcherry	36	11
Figure 9A-9C	Cav3.2 mcherry Cav3.1 -/-	28	9
	Cav3.2 mcherry	32	23
Figure 9E	Cav3.2 mcherry Cav3.1 -/-	8	6
Figure 9F	Cav3.2 mcherry Cav3.1 -/-	7	6
Figure 9G	Cav3.2 mcherry Cav3.1 -/-	28	9
Figure 9H, 9I	Cav3.2 mcherry Cav3.1 -/-	12	8
	Cav3.2 mcherry	32	23
	Cav3.2 Cre mcherry	5	4
Figure 9J	Cav3.2 mcherry Cav3.1 -/-	28	9

Supplementary Table 4. Neurons and mice used for statistical analysis in supplementary Figures.

Figure	Neuron type	Nb of neurons	Nb of mice
Sup. Fig. 3A, 3B	Unidentified	9	5
Sup. Fig. 3C, 3D	Unidentified	9	5
Sup. Fig. 3E, 3F	Unidentified	5	3
Sup. Fig. 4D	Unidentified	19	12
Sup. Fig. 4E	Unidentified	8	8
Sup. Fig. 5B	Unidentified	14; 16	25
Sup. Fig. 5C-5E	Unidentified	9; 14	18
Sup. Fig. 6C	Unidentified	134	56

Supplementary Table 5. Parameters of the firing patterns in Cav3.2-expressing neurons.

	Electrophysiological parameter				
**Firing patterns**	**Rheobase (pA)**	**Nb AP**	**1st Latency (ms)**	**Firing time (ms)**	**SD IeI (ms)**
**Single Spiking (8)**	61.9 ± 9.3	1.8 ± 0.2	317.6 ± 161.1	487.6 ± 224.6	83.8 ± 60.6
**Transient (7)**	82.1 ± 20.9	14 ± 5.7	23.2 ± 5.2	428.4 ± 158.7	16.8 ± 4.7
**Irregular tonic (15)**	72.1 ± 21.7	15.1 ± 1.8	40.9 ± 7.1	1705.8 ± 86.9	135.5 ± 48.4
**Regular tonic (14)**	35.4 ± 4.0	20.4 ± 2.1	64.0 ± 6.1	1891.9 ± 37	38.7 ± 11.7
**Delayed (30)**	39.7 ± 2.7	21.2 ± 1.9	169.5 ± 12.5	1872.9 ± 54.6	57.6 ± 11.2
**Gap (17)**	56.2 ± 5.4	13.2 ± 1.8	315.5 ± 37.2	1790.9 ± 99.6	111.7 ± 20.1

Data are expressed as mean ± SEM. Numbers of neurons are within brackets. Nb AP: number of action potentials elicited during a 2s-step current pulse, SD IeI, standard deviation of the intervals between action potentials elicited during a 2s-step current pulse.

Supplementary Table 6. Properties of the action potential in Cav3.2-expressing neurons.

	Electrophysiological parameter				
**Firing patterns**	**Threshold (mV)**	**Peak (mV)**	**AHP (mV)**	**hWidth (ms)**	**TTP (ms)**
**Single Spiking (8)**	-37.3 ± 3.4	13.0 ± 2.1	-46.8 ± 2.9	0.86 ± 0.10	2.6 ± 0.9
**Transient (7)**	-36.0 ± 2.0	17.8 ± 3.6	-46.1 ± 2.1	0.96 ± 0.11	1.8 ± 0.5
**Irregular tonic (15)**	-36.9 ± 1.6	12.1 ± 2.4	-47.2 ± 1.7	0.92 ± 0.08	2.2 ± 0.3
**Regular tonic (14)**	-37.7 ± 1.1	10.5 ± 2.1	-54.0 ± 0.9	0.74 ± 0.04	2.1 ± 0.3
**Delayed (30)**	-32.0 ± 0.7	10.0 ± 1.1	-49.7 ± 0.7	0.82 ± 0.05	1.9 ± 0.2
**Gap (17)**	-31.7 ± 0.9	13.8 ± 2.3	-51.0 ± 1.6	0.77 ± 0.02	1.9 ± 0.3

Data are expressed as mean ± SEM. Numbers of neurons are within brackets. AHP: afterhyperpolarization; hWidth: half-width of the action potential; TTP, time-to-peak.

Supplementary Table 7. Significant effects of Cav3.2 ablation on the excitability of LII neurons classified according to subthreshold properties.

	Cav3.2-mcherry	Cav3.2-Cre-mcherry	Mann Whitney test
**Rebound-exhibiting neurons**	**(31)**	**(5)**	
Peak (mV)	12.9 ± 1.6	18.8 ± 1.5	p=0.01
After-hyperpolarisation (mV)	-49.2 ± 1.3	-56.5 ± 1.4	p=0.002
Time-to-peak (ms)	2.57 ± 0.29	1.24 ± 0.4	p=0.03
Half-Width (ms)	0.87 ± 0.04	0.67 ± 0.04	p=0.006
Activity time (ms)	1390 ± 136	1826 ± 47	p=0.005
**Hyperpolarizing neurons**	**(29)**	**(8)**	
Peak (mV)	10.6 ± 1.1	17.6 ± 3.4	p=0.02
Activity time (ms)	1753 ± 84	1928 ± 201	p=0.04
**Passive neurons**	**(31)**	**(21)**	
Threshold (mV)	-32.2 ± 0.7	-29.5 ± 0.9	p=0.04

Data are expressed as mean ± SEM. Numbers of neurons are within brackets. Data are shown when $p<0.05$, as a result of the Mann Whitney test between the two populations of Cav3.2-mcherry positive neurons.
